# A glass bead semi-hydroponic system for intact maize root exudate analysis and phenotyping

**DOI:** 10.1186/s13007-022-00856-4

**Published:** 2022-03-05

**Authors:** Martha G. Lopez-Guerrero, Peng Wang, Felicia Phares, Daniel P. Schachtman, Sophie Alvarez, Karin van Dijk

**Affiliations:** 1 Biochemistry Department, University of Nebraska—Lincoln, Lincoln, NE 68588 USA; 2 Department of Agronomy and Horticulture, University of Nebraska—Lincoln, Lincoln, NE 68588 USA; 3 Center for Plant Science Innovation, University of Nebraska—Lincoln, Lincoln, NE 68588 USA; 4 Nebraska Center for Biotechnology, University of Nebraska—Lincoln, Lincoln, NE 68588 USA

**Keywords:** Maize, Corn, *Zea mays*, Root exudates, Hydroponics, Glass beads, Targeted metabolomics, LC–MS/MS, GC–MS, Mass spectrometry, MCX-SPE, Amino acids, Phytohormones, Sugars, CaCl_2_

## Abstract

**Background:**

Although there have been numerous studies describing plant growth systems for root exudate collection, a common limitation is that these systems require disruption of the plant root system to facilitate exudate collection. Here, we present a newly designed semi-hydroponic system that uses glass beads as solid support to simulate soil impedance, which combined with drip irrigation, facilitates growth of healthy maize plants, collection and analysis of root exudates, and phenotyping of the roots with minimal growth disturbance or root damage.

**Results:**

This system was used to collect root exudates from seven maize genotypes using water or 1 mM CaCl_2_, and to measure root phenotype data using standard methods and the Digital imaging of root traits (DIRT) software. LC–MS/MS (Liquid Chromatography—Tandem Mass Spectrometry) and GC–MS (Gas Chromatography—Mass Spectrometry) targeted metabolomics platforms were used to detect and quantify metabolites in the root exudates. Phytohormones, some of which are reported in maize root exudates for the first time, the benzoxazinoid DIMBOA (2,4-Dihydroxy-7-methoxy-1,4-benzoxazin-3-one), amino acids, and sugars were detected and quantified. After validating the methodology using known concentrations of standards for the targeted compounds, we found that the choice of the exudate collection solution affected the exudation and analysis of a subset of analyzed metabolites. No differences between collection in water or CaCl_2_ were found for phytohormones and sugars. In contrast, the amino acids were more concentrated when water was used as the exudate collection solution. The collection in CaCl_2_ required a clean-up step before MS analysis which was found to interfere with the detection of a subset of the amino acids. Finally, using the phenotypic measurements and the metabolite data, significant differences between genotypes were found and correlations between metabolites and phenotypic traits were identified.

**Conclusions:**

A new plant growth system combining glass beads supported hydroponics with semi-automated drip irrigation of sterile solutions was implemented to grow maize plants and collect root exudates without disturbing or damaging the roots. The validated targeted exudate metabolomics platform combined with root phenotyping provides a powerful tool to link plant root and exudate phenotypes to genotype and study the natural variation of plant populations.

**Supplementary Information:**

The online version contains supplementary material available at 10.1186/s13007-022-00856-4.

## Background

Root exudates are known to play an important role in plant biological processes. They contribute to the interaction between plant roots and the surrounding soil by increasing nutrient and water availability [1, 2], and by modulating root interactions with the microbiome and nearby plants to maintain a sustainable environment for growth [3, 4]. The composition of root exudates is diverse, ranging from sugars to organic acids, flavonoids, phytosiderophores, phenolics, amino acids, phytohormones, and high molecular weight compounds such as proteins and polysaccharides [5–7]. Some of these compounds are unique to specific plant taxa, like sorgoloeone which is a lipophilic compound mainly found in *Sorghum* spp.[8, 9]*.* Many compounds in exudates have been shown to contribute to shaping the microbial community by recruiting microbes, such as fungi [10] and bacteria with different metabolic capacities [11–13]. Knowledge of how exudates interact with specific beneficial microbes in the rhizosphere may eventually be used to enhance crop production and crop tolerance to stress [14–16]. Although to date few studies have focused on modulating exudation to improve crop yield, recent reports showed that regulation of the AtALMT1 aluminum-activated root malate transporter, responsible for malate exudation, affects crop tolerance to aluminum toxicity, low phosphorus availability, and drought stress [14].

The study of plant root exudates is complicated by the vast diversity of the metabolites that different plant species produce and the growth of roots in soil containing a large variety of microbes. While we know a great deal about root exudate composition in some species like rice [17–19] and *Arabidopsis* [20–22], the vast array of metabolites produced by plants makes it difficult to infer the composition of these root exudates to all plant species. Moreover, multiple approaches to collect root exudates have been described, each with their own limitations. There are only a few examples of attempts to collect exudates in situ, either in field soils [23] or in greenhouse soils [24]. It is well documented that some root exudates are metabolized by the soil microbial communities, and this along with compounds secreted by microbes into the rhizosphere confounds the analysis of root exudates in soil [5]. An alternative approach has been to grow plants in soil or sand and to collect exudates after removal and washing of soil from roots [13, 25] which also confounds the analysis due to the large disturbance of the roots. In order to remove the confounding factor of soil microbes, hydroponics or supported hydroponics using substrates such as gels, glass beads, and vermiculite, which provide sterile or semi-sterile systems, have been used to grow plants and collect exudates [5, 26]. Glass beads used in semi-hydroponic systems are re-usable, easily sterilized and potentially provide an inert substrate that partially simulates the natural mechanical impedance that roots experience in soil [27]. The glass bead system reported on here also allows for collection without mechanical disturbance of the roots. In maize, the use of such a system resulted in increased root exudation but reduced root elongation due to the impedance imposed by the glass beads when compared to plants grown in hydroponics without glass beads [27, 28].

In addition to the impact of the plant growth substrate on root exudation, the choice of exudate collection solution is important and may introduce experimental artefacts. Root exudates are most commonly collected with deionized water [29, 30], or CaCl_2_ solutions [31–33]. In some cases, NaN_3_, MES-KOH buffer [34] or culture media [18, 20, 22] have been used. Although some studies suggest that water may be suitable for exudate collection [34, 35], other studies have found that the use of water can increase the exudation of specific compounds. For example, in rice [36] and *Lupinus albus* [34], amino acids and organic acids levels, amongst others, are higher in exudates when collected in deionized water [34, 36]. It has been suggested that the use of water for collection is responsible for leakage of compounds because of the irreversible loss of root membrane integrity [34]. Presence of salts like CaCl_2_ at a concentration of at least 100 µM prevents cell damage [37, 38].

Here we report on a plant growth system designed to collect exudates from undisturbed maize roots as well as their phenotypes to facilitate comparisons between genotype exudate profiles and other plant traits. We designed the growth system with a substrate that mimics some of the structural features of soils, yet lacks the microbial and chemical soil complexity, to reliably collect and analyze root exudate composition and phenotype roots with minimal root disturbances. To determine if the system was reliable for the comparison of root exudate composition, seven maize inbred genotypes from the Buckler-Goodman diversity panel were grown in this glass bead supported hydroponics system. Exudates were collected and analyzed using Milli-Q (MQ) water or CaCl_2_ as exudate collection solutions. We used targeted metabolomics to detect a wide range of compounds, including phytohormones, the benzoxazinoid DIMBOA, amino acids [including GABA (gamma-aminobutyric acid)], and sugars. Our results suggest that the use of water to collect exudates may be responsible for leakage of some metabolites. In addition, using this system we detected significant differences in exudate concentrations of amino acids and phenotypic traits between genotypes and enabled the correlation between metabolites and phenotypic traits.

## Results

### Glass bead supported semi-hydroponics system

**Fig. 1:**
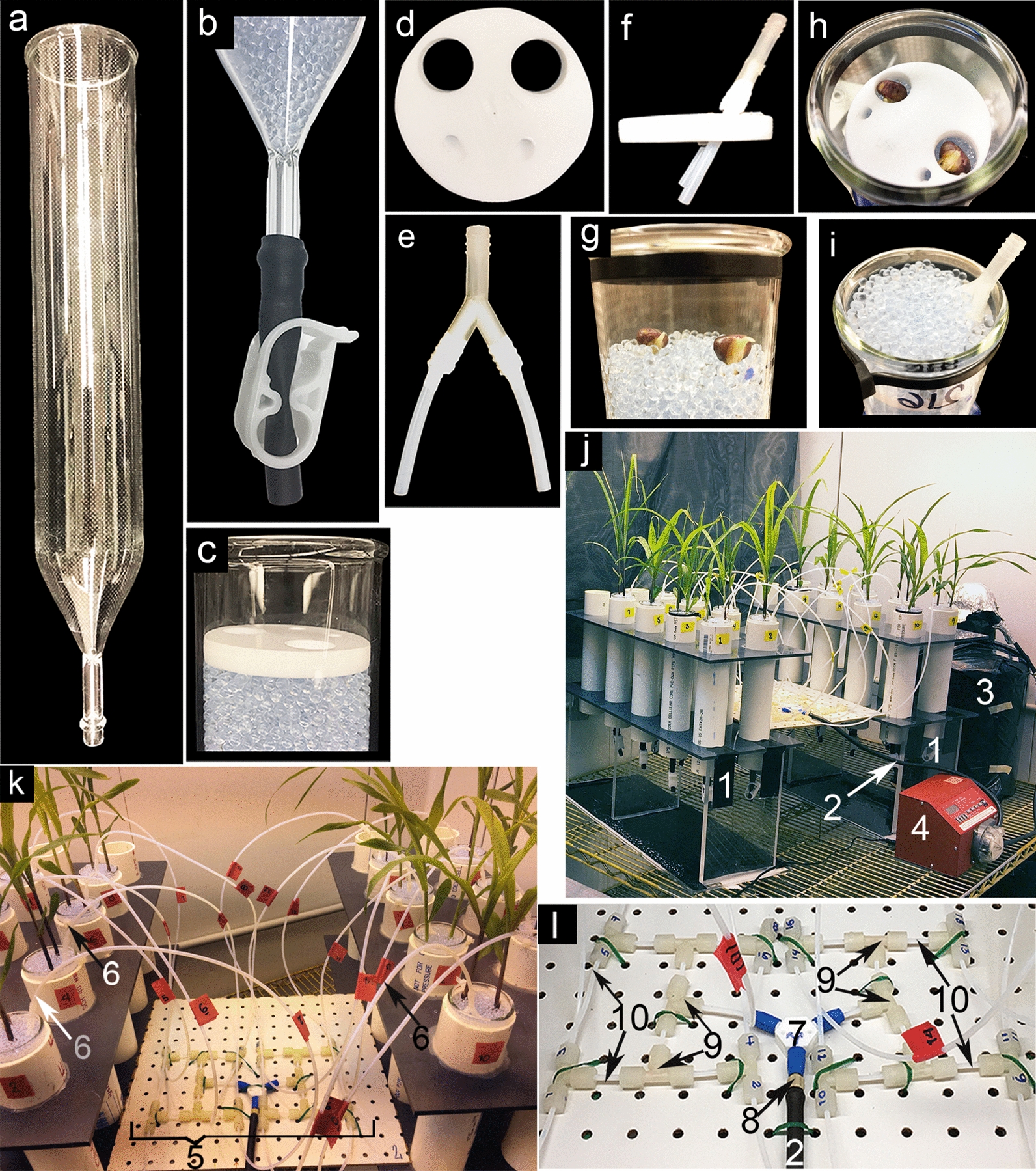
Glass Bead semi-hydroponic system. **a** Glass tube. **b** Drain glass tube connected to 3 cm Viton tubing and closed with an acetal clamp. **c** Top of the glass tube filled with glass beads covered with a Teflon lid. **d** Details of the Teflon lid showing the four perforations. **e** “Y connector” attached to 4 cm Teflon tubing and sealed with Teflon tape. **f** “Y connector” inserted in the Teflon lid, showing the diagonal angle to water the plants. **g** Germinated seeds placed on top of the glass beads. **h** Planted seeds covered by the Teflon lid after planting. **i** Glass tube with planted seeds covered by glass beads and with the “Y connector” inserted. **j** Plants growing in the glass tubes in a growth chamber (1. Rack tubes, 2. Viton line, 3. Glass carboy containing Hoagland solution for watering plants. 4. Peristaltic pump). **k** Details of the watering system (5. Branched pipe 2 × 8, 6. Outlet lines inserted in the “Y connectors”). **l** Details of the branched pipe (7. 3-way connector, 8. Barbed adaptor, 9. Compression fittings, 10. Teflon tubing connecting the compression fittings)

We designed a glass bead supported semi-hydroponics system to grow plants described in detail in the methods section (Fig. 1). To minimize microbial contamination, all growth and watering components can be autoclaved, and watering is done in a manner that maintains a semi-sterile environment, as sterile nutrient solution is pumped via individual tubes to each glass tube without solution recirculation. The plants were not disturbed prior and during exudate collection since the system enables collection of exudates in the same container in which the plants are grown. Given that the root system of some genotypes outgrew the glass tubes if grown for more than 15 days, we collected root exudates 14 days after planting with 1 mM CaCl_2,_ and 15 days after planting with MQ. No differences in plant growth (data not shown) were observed when different-sized glass beads (1 mm, 2 mm, or 3 mm) were tested as growth support, hence, we used 3 mm glass beads due to their lower cost. The system is scalable and can be adapted to accommodate various experimental designs. For example, each glass tube can support the growth of one to two plants, and each tube rack can be used to grow either different genotypes or a different number of replicates of the same genotype.

To determine if it was feasible to use the system for root exudate analyses and comparisons between genotypes, we designed an experiment with seven different maize (*Zea mays* L.) genotypes, using at least three replicate tubes containing two plants, and analyzed the composition of root exudates collected with the two commonly used exudate collection solutions, 1 mM CaCl_2_ and MQ. We also compared the root phenotypes. At the end of the experiment the roots and shoots looked healthy with no apparent signs of stress. Additional files 1 and 2 show representative pictures of the root system and aerial part of each genotype, respectively. The root morphology of two to four genotypes grown in hydroponics, soil, and sand, were compared to those grown in the glass bead semi-hydroponic system to visualize the differences in root morphology due to growth substrate (Additional file 3a, b). The comparison highlights that the similarities or differences in root morphology between each system was genotype dependent and therefore no clear generalizations could be derived, except that unlike roots in hydroponic systems, roots in the glass bead semi-hydroponic system formed hairs (Additional file 3c).

### Plant root phenotyping of the tested genotypes

**Fig. 2:**
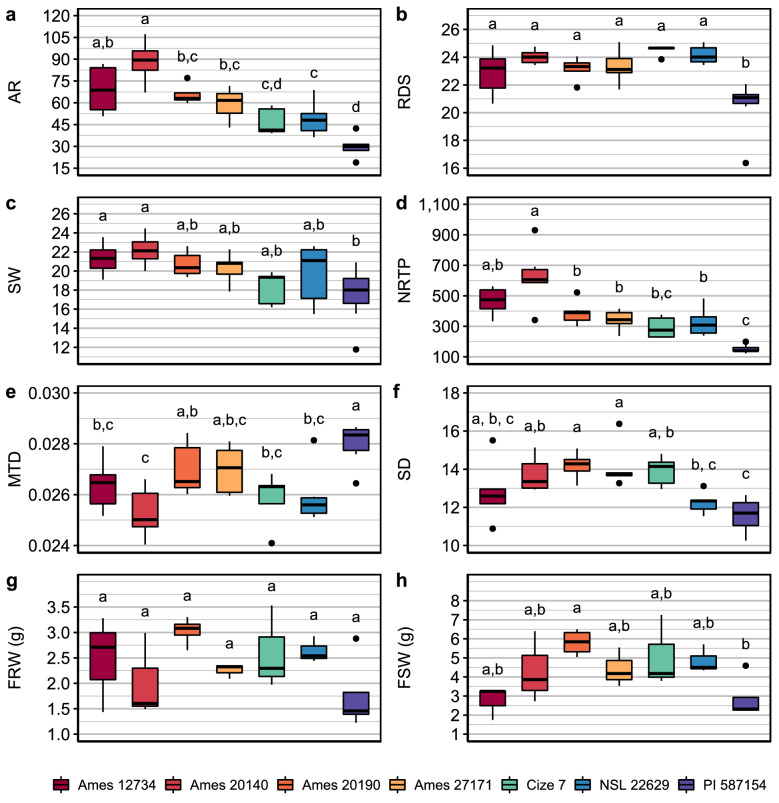
Differences in root and shoot phenotypic traits between genotypes. **a** Root area (AR), number of foreground pixels belonging to the root system. **b** Rooting depth skeleton (RDS), describes the longest root length. **c** Skeleton width (SW), which was calculated from the medial axis of the root system. **d** Number of root tips paths (NRTP), which is the overall number of tips detected in the image. **e** Mean tip diameter (MTD), **f** Stem diameter (SD), derived from the medial axis. **g** Fresh root weight (FRW). **h** Fresh shoot weight (FSW). Statistical differences detected by All pairs Tukey–Kramer HSD (honest significant difference), α = 0.05. Measurements with different letters within each graph are significantly different. Traits obtained by DIRT using the scanned root images, panels **a**–**f**, are represented with arbitrary units given by DIRT. Roots were scanned individually, while root and shoot weight represent the average of the total root or shoot weight divided by the number of plants grown in a single glass tube used to collect exudates. Number of samples used to obtain DIRT parameters AR, RDS, SW, NRTP, MTD, SD: Cize 7 n = 5; Ames 12734, Ames 20140, Ames 27171, NSL 22629 n = 6; Ames 20190 n = 7; PI 57154 n = 8. Number of samples for FRW and FSW: Cize 7, Ames 12734, Ames 20140, Ames 27171, NSL 22629 n = 3; Ames 20190, PI 57154 n = 4. Boxplot: Box, interquartile range (IQR); line inside the box, median; end of the box, upper (Q3) and lower (Q1) quartiles; dots beyond the extreme lines show potential outliers

The roots from the genotypes used in this study were characterized phenotypically. In addition to measuring root and shoot fresh weights, roots were scanned, and the images were analyzed using the Digital imaging of root traits (DIRT) software [39]. We found significant differences between at least two genotypes for all analyzed phenotypes except for fresh root weight (FRW) (Fig. 2). The genotype PI 587154 had the lowest values in all phenotypes except for the mean tip diameter (MTD), while Ames 20140 was at the opposite spectrum with the highest values for all except the mean tip diameter. The genotypes selected for this study showed a broad range of natural variation in phenotypes.

### Characterization of metabolites in root exudates collected with CaCl_2_ or MQ

**Fig. 3:**
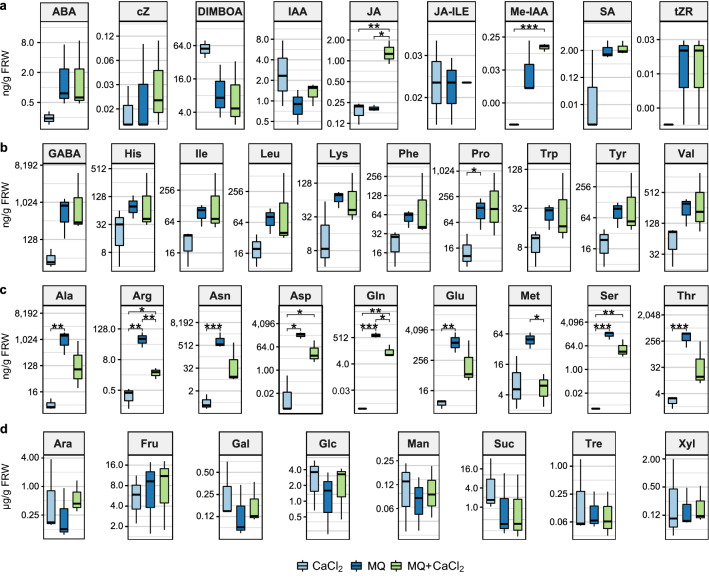
Root exudates collected with 1 mM CaCl_2_ or MQ in the genotype Ames 20140. Blue bars: analysis of exudates collected with MQ or CaCl_2_. Green bars: analysis of the effect on the recovery of exudates after a clean-up step using MCX-SPE cartridges to remove salts from the samples collected with  MQ and added CaCl_2_. **a** Phytohormones detected (ABA, cZ, JA, JA-Ile, Me-IAA, tZR, SA, IAA). **b** Amino acids, group 1, detected in MQ and CaCl_2_ with no differences (GABA, His, Ile, Leu, Lys, Phe, Pro, Trp, Tyr, Val). **c** Amino acids, group 2, detected in low concentration in CaCl_2_ (Ala, Arg, Asn, Asp, Gln, Glu, Met, Ser, Thr). **d** Sugars (Ara, Fru, Gal, Glc, Man, Suc, Tre, Xyl). Statistical differences detected with a T-test, each pair, only significant differences shown *p ≤ 0.05, **p ≤ 0.01. Ames 20140, n = 3. Boxplot: Box, interquartile range (IQR); line inside the box, median; end of the box, upper (Q3) and lower (Q1) quartiles; dots beyond the extreme lines show potential outliers

A panel of phytohormones, the benzoxazinoid DIMBOA, amino acids, and sugars were measured in the root exudates collected either with 1 mM CaCl_2_ or MQ (Fig. 3, Additional files 4, 5, 6, and 7). Phytohormones and amino acids were detected in the exudates using two separate targeted LC–MS/MS (Liquid Chromatography-Tandem Mass Spectrometry) approaches in all seven genotypes, while sugars were quantified using a targeted GC–MS (Gas Chromatography–Mass Spectrometry) on four of the seven genotypes. Out of the 23 phytohormones in the panel (as detailed in the Methods section), eight were detected and quantified consistently in all samples: ABA (abscisic acid), cZ (cis-zeatin), JA (jasmonic acid), JA-Ile (jasmonyl-isoleucine), Me-IAA (methyl indole-3-acetic acid), tZR (trans-zeatin riboside), SA (salicylic acid) and, IAA (indole-3-acetic acid). The benzoxazinoid DIMBOA was also detected in all samples. The following 19 amino acids were detected and quantified in all samples; His (histidine), Ile (isoleucine), Leu (leucine), Lys (lysine), Met (methionine), Phe (phenylalanine), Pro (proline), Trp (tryptophan), Tyr (tyrosine), Val (valine), GABA, Ala (alanine), Arg (arginine), Asn (asparagine), Asp (aspartic acid), Gln (glutamine), Glu (glutamic acid), Ser (serine) and, Thr (threonine). Finally, eight out of ten sugars were detected and quantified in four genotypes: Ara (arabinose), Fru (fructose), Gal (galactose), Glc (glucose), Man (mannose), Suc (sucrose), Tre (trehalose) and Xyl (xylose), whereas lactose and raffinose were not detected in root exudates.

To compare the exudate composition between genotypes as well as between the two different exudate collection solutions, we normalized the phytohormone, amino acid and sugar concentrations per gram of fresh root weight (FRW) for each genotype. The detailed results per genotype are shown in the Additional files 4, 5, 6, and 7. The results obtained for the Ames 20140 genotype exemplify those found in the other genotypes. This genotype was selected randomly (Fig. 3). We did not find significant differences in the phytohormones between root exudates collected in 1 mM CaCl_2_ or MQ (pairwise T-test, α = 0.05) (Fig. 3a; Additional file 4). On the other hand, for all amino acids analyzed, concentrations were noticeably higher in exudates collected with MQ compared to those collected with 1 mM CaCl_2_ (Fig. 3b, c; Additional files 5 and 6). However, the difference in concentrations was statistically significant only for a subset of the amino acids. Except for Pro, the differences were not statistically significant for the amino acids designated as group 1 AA: GABA, His, Ile, Leu, Lys, Phe, Pro, Trp, Tyr and, Val (pairwise T-test, α = 0.05) (Fig. 3b; Additional file 5a, b). However, except for Met, the differences were statistically significant for the amino acids designated as group 2 AA: Ala, Arg, Asn, Asp, Gln, Glu, Met, Ser, and Thr (Fig. 3c) for most genotypes (Additional file 6). Group 2 AA amino acids were all detected at low levels in 1 mM CaCl_2_, close to the limits of detection when compared with levels obtained with MQ. The group designations are further described below. We did not find statistically significant differences between the concentration of sugars in the exudates collected with 1 mM CaCl_2_ and MQ in any of the genotypes tested (pairwise T-test, α = 0.05) (Fig. 3d; Additional file 7a).

### Recovery test of the metabolites from CaCl_2_ collection using standards

The protocol to analyze the phytohormones and amino acids from the samples collected with 1 mM CaCl_2_ includes an SPE (solid phase extraction) cartridge clean-up step to remove the salts which would interfere with the LC–MS/MS assay. This step is not needed when the samples are collected in MQ. The SPE cleaning protocol uses an MCX cartridge (mixed mode strong Cation-eXchange), with both reverse phase and cationic exchange retentive properties, selective for bases (pKa 2–10) and hydrophobic compounds. This cleaning and concentrating procedure has been used previously to study amino acids content in root exudates [32, 40]. However, because of the significant differences found in the amino acids in the exudates collected in 1 mM CaCl_2_ and MQ, we analyzed the impact of the MCX-SPE step on the recovery of the metabolites analyzed. To differentiate between the impact of the MCX-SPE step and the effect of the solution used to collect exudates, we first analyzed the recovery of phytohormones and amino acids using a mixture of standards at known concentrations resuspended in either 1 mM CaCl_2_ or MQ but this time both were processed through the MCX-SPE cartridges (labeled “SPE-CaCl_2_” and “SPE-MQ”, respectively). The recovery was evaluated comparing the results against a control sample of standards resuspended in MQ but not cleaned-up with the MCX-SPE.

The percentage of recovery before normalization of the phytohormones was similar for the SPE-CaCl_2_ and SPE-MQ treatments, ranging from 39 to 99% for the SPE-CaCl_2_ sample and 33–98% for the SPE-MQ (Table 1). Although the recovery was not 100%, the coefficient of variation (CV) was low, below 10% for most phytohormones, which shows reproducibility and robustness of the results when the MCX-SPE step is done for phytohormones analysis. The phytohormones with the best recovery rate were SA and ABA. DIMBOA had the lowest recovery rate and a CV of 20%. After normalization, using the internal standards spiked in the samples, the recovery levels were close to 100%, with a few exceptions. The exceptions are due to the difference in recovery of the internal standard used to normalize the data from the standard. For example, cZ and D5tZ (D5-trans-zeatin) had a recovery rate of 68 and 81%, respectively, in SPE-MQ. Because the internal standard had a better recovery than the standard, after normalization the rate for cZ stayed below 100% with 83% recovery. In the opposite scenario, SA and D4SA (D4-salicylic acid) with recoveries of 99 and 81%, respectively, in SPE-CaCl_2_ had a recovery of 123% after normalization.

**Table 1:** Recovery percentages of the phytohormones after MCX-SPE cleaning

	% recovery before normalization		% recovery after normalization		CV in %	
	SPE-CaCl2	SPE-MQ	SPE-CaCl2	SPE-MQ	SPE-CaCl2	SPE-MQ
ABA	84	85	105	104	2.8	5.1
D6ABA	80	82	100	100	4.4	1.8
cZ	65	68	88	83	1.5	2.7
D5tZ	74	81	100	100	4.4	7.4
tZR	62	65	106	105	3.9	3.2
D5tZR	59	62	100	100	8.8	8.1
SA	99	98	123	117	4.7	6
D4SA	81	84	100	100	7.2	5.6
IAA	51	56	110	102	4.8	2.2
Me-IAA	44	33	95	61	6.8	9.6
D5IAA	47	55	100	100	12.1	5
JA	52	52	104	104	3.2	8.5
JA-Ile	74	74	142	181	5.2	3.3
D2JA	53	41	100	100	12.6	12.2
DIMBOA	39	56	55	75	19.5	20

Recoveries were calculated before and after normalization using the internal standards *D6ABA (D6-abscicic acid)* for ABA*, D5tZ (D5-trans-zeatin)* for cZ*, D5tZR (D5-trans-zeatin riboside)* for tZR, *D5IAA (D5-indole-3-acetic acid)* for IAA and Me-IAA, *D2JA (D2-jasmonic acid)* for JA and JA-Ile, and the average of all the internal standard (IS) for DIMBOA. CV (coefficient of variation) is also included

The recovery rates of the amino acids were more variable than those of phytohormones (Table 2). For the analysis, we divided amino acids into two groups based on the differences in their recovery in CaCl_2_ and MQ, recovery percentage and CV. In group 1 AA (GABA, His, Ile, Leu, Lys, Phe, Pro, Trp, Tyr, Val and the internal standard NVa (norvaline), we found no difference between SPE-CaCl_2_ and SPE-MQ treatments, and all have a low CV (Table 2a). The CaCl_2_ in solutions reduced the recovery of the amino acids, GABA, Pro and Val but in a reproducible way, with a CV < 7%. The data from group 2 AA (Ala, Arg, Asn, Asp, Gln, Glu, Met, Ser, and Thr) showed not only that the MCX-SPE clean-up step is responsible for higher variability (higher CV), but also shows that the presence of CaCl_2_ exacerbates the poor selectivity of the MCX sorbent for these amino acids (Table 2b). The recovery and CV data from this recovery test correlate with the results previously observed with the amino acids that were detected at low abundance in the root exudate samples collected with 1 mM CaCl_2_ (Fig. 3c, Additional file 6).

**Table 2:** Recovery percentages of amino acids after MCX-SPE cleaning

a. Group 1 of amino acids detected in both 1 mM CaCl_2_ and MQ						
	% recovery before normalization		% recovery after normalization		CV in %	
	SPE-CaCl_2_	SPE-MQ	SPE-CaCl_2_	SPE-MQ	SPE-CaCl_2_	SPE-MQ
GABA	27.5	79.8	35.4	106.5	5.8	6.5
His	79.7	82.4	102.6	109.5	9.5	7.2
Ile	74.3	79.8	95.8	106.2	2.3	5.2
Leu	80.7	78.8	104	104.8	1.6	3.5
Lys	97.1	99	125.1	131.7	3.9	6.3
Phe	84	76.3	108.2	101.5	1.1	2.4
Pro	37.8	81.7	48.7	108.6	6.7	4.5
Trp	53.2	49.3	68.3	65.3	25.4	19.2
Tyr	77.6	82.3	99.9	109.5	2.4	4.2
Val	60	79.9	77.3	106.3	4.7	3.7
Nva	77.7	75.3	–	–	3.1	5.9

b. Group 2 of amino acids detected in both 1 mM CaCl_2_ and MQ						
	% recovery before normalization		% recovery after normalization		CV in %	
	SPE-CaCl_2_	SPE-MQ	SPE-CaCl_2_	SPE-MQ	SPE-CaCl_2_	SPE-MQ
Ala	5.7	37	7.4	49.3	29	13.4
Arg	1.3	24	1.6	32.5	69	33.7
Asn	1.6	5.4	2.1	7.3	15.9	30.2
Asp	1.7	8.4	2.3	11.2	26.3	37.8
Gln	0.9	18.1	1.2	24.1	19	7
Glu	1.8	21.8	2.3	29.1	12.5	9.8
Met	15.6	29.7	20.2	39.1	40.4	25.2
Ser	4.7	12.6	6	16.2	86	110.6
Thr	1.7	14.1	2.3	18.8	56.9	18.2

Recoveries were calculated before and after normalization using the internal standard Nva (norvaline). CV (coefficient of variation) is also included

### Effect of CaCl_2_ and the MCX-SPE step on the recovery of phytohormones and amino acids from maize root exudates

CaCl_2_ and the MCX-SPE step impacted the recovery of standards so therefore we tested their impact on the recovery of complex mixtures of root exudate metabolites. To do so we used an aliquot of the exudates collected with MQ, added CaCl_2_ to the same final concentration as samples collected with 1 mM CaCl_2_, desalted the samples by MCX-SPE, and analyzed the samples by LC–MS/MS for phytohormone and amino acid concentrations. The samples were designated “MQ + CaCl_2_” and the results were compared to those obtained for the exudates collected in 1 mM CaCl_2_ and MQ (Fig. 3, Additional files 4, 5, and 6). Similar to our findings with the standards, the phytohormones concentrations in MQ and MQ + CaCl_2_ exudates were similar in that significant differences were not observed between the two types of samples for most of the measured phytohormones (pairwise T-test, α = 0.05) (Fig. 3a, Additional file 4). This confirmed that the MCX-SPE step did not alter the detection of most of the phytohormones. Similar results were found for group 1 AA (GABA, His, Ile, Leu, Lys, Phe, Tyr, Trp, Pro and Val) (Fig. 3b, Additional file 5). In contrast adding CaCl_2_ to the MQ samples, followed by MCX-SPE of the exudates, resulted in significant losses of amino acids from group 2 (Ala, Arg, Asn, Asp, Met, Gln, Glu, Ser, and Thr) (Fig. 3c, Additional file 6).

Finally, to determine if CaCl_2_ affected the sugar analysis by GC–MS, aliquots from root exudates collected in MQ from a couple of genotypes were analyzed after the addition of CaCl_2_ at the same concentration as the root exudates collected in 1 mM CaCl_2_ (labeled as “MQ + CaCl_2_”). No significant differences were found in sugars concentrations between the MQ and MQ + CaCl_2_ samples_,_ confirming that CaCl_2_ did not interfere with the GC–MS analysis of sugars (Fig. 3d, Additional file 7).

Our data show that the MCX-SPE step does not affect the recovery of most phytohormones, and half of the amino acids tested but is responsible for losses of some of the amino acids, making the method using CaCl_2_ as an exudate collection solution problematic for the analysis of the amino acids Ala, Arg, Asn, Asp, Met, Gln, Glu, Ser and Thr.

### Overall impact of the exudate collection in MQ and CaCl_2_

**Fig. 4:**
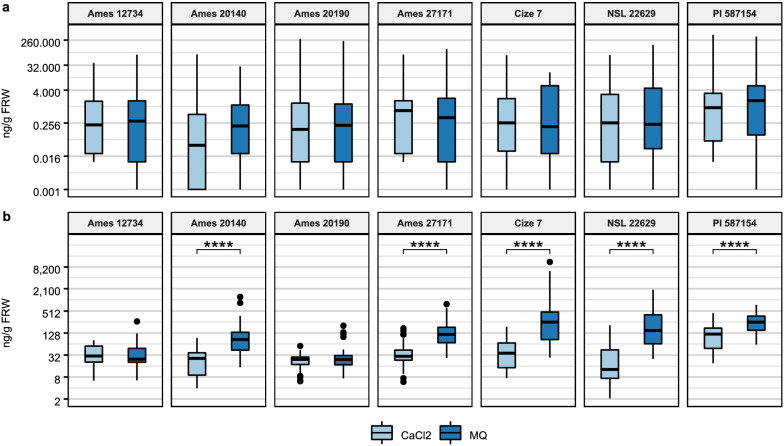
Global differences in phytohormones, DIMBOA, and amino acids in exudates collected from seven genotypes with 1 mM CaCl_2_ and MQ. All the phytohormones and all the amino acids detected were combined in one group each to understand globally the differences in the recovery of each group of metabolites. **a** Total amount of all phytohormones detected (ABA, cZ, JA, JA-Ile, Me-IAA, tZR, SA, IAA) and DIMBOA. Cize 7, Ames 12734, Ames 20140, Ames 27171, 22629 n = 27 (3 samples per genotype; 8 phytohormones and DIMBOA); Ames 20190, PI 57154 n = 36 (4 samples per genotype; 8 phytohormones and DIMBOA) **b** Total amount of all amino acids detected in MQ and CaCl_2_ (GABA, His, Ile, Leu, Lys, Phe, Pro, Trp, Tyr, Val). Cize 7, Ames 12734, Ames 20140, Ames 27171, NSL 22629 n = 30 (3 samples per genotype; 10 amino acids). Ames 20190, PI 57154 n = 40 (4 samples per genotype; 10 amino acids). Statistical differences detected with a T-test, each pair, only significant differences shown. ****p ≤ 0.0001. Boxplot: Box, interquartile range (IQR); line inside the box, median; end of the box, upper (Q3) and lower (Q1) quartiles; dots beyond the extreme lines show potential outliers

We excluded the amino acids which incurred a loss from the MCX-SPE step and compared the impact of the solution used to collect root exudates based on the concentration of each compound individually. We found that, except for some phytohormones in specific genotypes (DIMBOA and Me-IAA in Cize 7, and tZR in NSL 22629, Additional file 4) there were no statistical differences between the concentrations in MQ and CaCl_2_. However, when all three treatments were compared, 1 mM CaCl_2_, MQ and MQ + CaCl_2,_ there was a trend of higher exudate concentration of the compounds in the samples collected with MQ and MQ + CaCl_2_ compared to the concentrations in exudates collected with 1 mM CaCl_2_, suggesting that collection of exudates with water may lead to higher levels of root exudation. To better understand the impact of the collection solution on exudation, we reanalyzed the data by combining concentrations of all phytohormones detected and combining the amino acid concentrations from group 1 AA as total amount in ng/g FRW for each genotype. As shown in Fig. 4, there was no significant difference in the phytohormone concentrations between exudates collected with 1 mM CaCl_2_ or MQ in any of the genotypes (Fig. 4a). However, in five out of the seven genotypes tested, we detected a significant difference in the combined amino acid concentration (Fig. 4b). The same tendency was found when the MQ + CaCl_2_ treatment was compared against 1 mM CaCl_2_ (data not shown). These data show that the amino acid exudation tends to be higher when MQ is used as the collection solution (2.8 times higher in PI 587154 and 14.4 times higher in Cize 7).

### Correlation between metabolic and genomic diversity

**Fig. 5:**
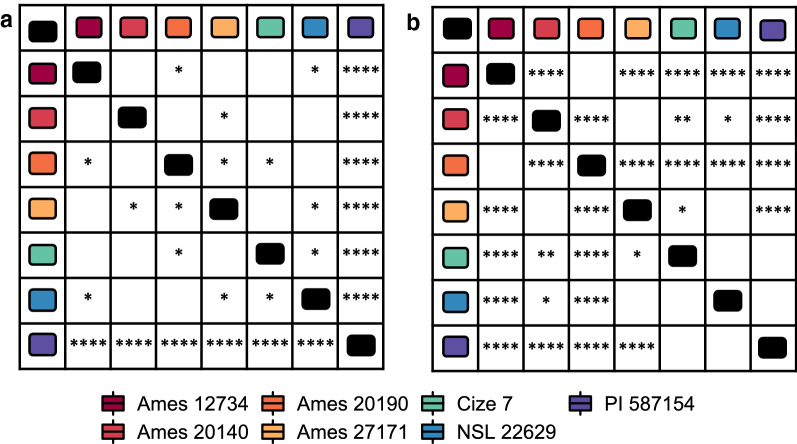
Genotypic differences in the total concentration of amino acids detected in exudates collected with MQ and 1 mM CaCl_2_ **a** Pairwise comparisons between genotypes for total concentrations of amino acids detected in exudates collected with CaCl_2_. **b** Pairwise comparisons between genotypes for total concentrations of amino acids detected in exudates collected in MQ. Statistical differences detected by a T-test each pair, only significant differences shown, *p ≤ 0.05, **p ≤ 0.01, ***p ≤ 0.001, ****p ≤ 0.0001. Cize 7, Ames 12734, Ames 20140, Ames 27171, NSL 22629 n = 3; Ames 20190, PI 57154 n = 4

Since our aim was to develop a semi-sterile root exudate collection system that mimics soil, and allows for exudate composition comparisons between genotypes, we analyzed if this system would allow for determination of relationships between genotype, exudate profile and other traits, and thus provide a screening method to determine natural variation of populations. To do this we analyzed the total concentrations of phytohormones and group 1 AA in the genotypes. Significant differences were only found when the total amino acid content was compared between genotypes with at least three replicates per genotype (Additional file 8). Pairwise comparisons (pairwise T-test, α = 0.05) were performed between genotypes comparing the total concentrations of the amino acid content when exudates were collected with 1 mM CaCl_2_ or MQ. When we considered the CaCl_2_ exudates, we found significant differences (p ≤ 0.0001) between PI 587154 and all other genotypes (Fig. 5a), consistent with the results from the phenotypic traits (Fig. 2). Moreover, when we analyzed the MQ exudates, we found additional significant differences between several other genotypes (Fig. 5b), suggesting that the two approaches provide sensitive and reliable methods to distinguish genotype-dictated exudate differences with some contrasting results.

**Fig. 6:**
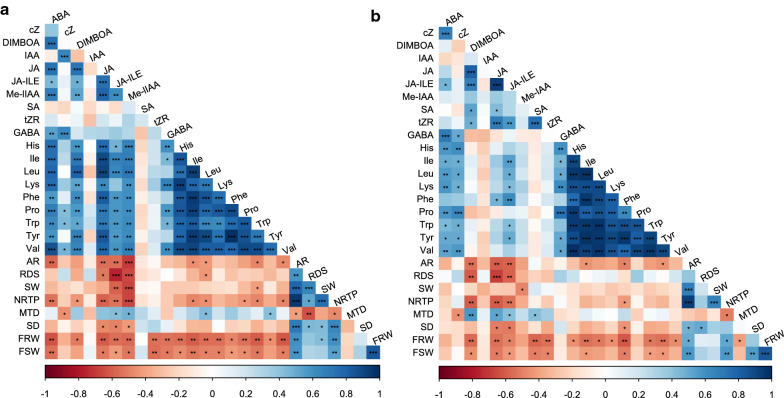
Correlation between metabolites and phenotypic characteristics. **a** Correlation between metabolites collected with CaCl_2_ and root and shoot phenotypic characteristics. **b** Correlation between metabolites collected with MQ and root and shoot phenotypic characteristics. AR (Root area), RDS (Rooting depth skeleton), SW (Skeleton width), NRTP (Number of root tips paths), MTD (Mean tip diameter), SD (Stem diameter), FRW (Fresh root weight), FSW (Fresh shoot weight). Pearson correlation. Blue, positive correlation. Red, negative correlation. White, no correlation. Significant correlations shown *p ≤ 0.05, **p ≤ 0.01, ***p ≤ 0.001, ****p ≤ 0.0001. Clustering: Ward method. Number of samples used to obtain DIRT parameters AR, RDS, SW, NRTP, MTD, SD: Cize 7 n = 5, Ames 12734, Ames 20140, Ames 27171, NSL 22629 n = 6; Ames 20190 n = 7, PI 57154 n = 8. Number of samples of FRW and FRW: Cize 7, Ames 12734, Ames 20140, Ames 27171, NSL 22629 n = 3; Ames 20190, PI 57154 n = 4

To determine if this method allows for detection of correlations between exudate content and the plant phenotypes measured, we performed a Pearson correlation analysis between all the metabolites (except for sugars) and phenotypic traits gathered from the 7 maize genotypes (Fig. 6). As would be expected, we found strong positive correlations (p ≤ 0.001) between the amino acids collected in both MQ and CaCl_2_ exudates. We also found strong negative correlations (p ≤ 0.01) between the metabolites and several root phenotypic traits. Root and shoot fresh weight (FRW and FSW, respectively) negatively correlated with most of the metabolites. In addition, we found significant negative correlations between the root phenotypic traits (AR, SW, RDS and NRTP) and three phytohormones, JA, JA-Ile and Me-IAA; this means that with an increased root area or increased number of root tips, there is a decreased level of these three phytohormones in the root exudates. It is worth noting that overall, there were stronger correlations observed with the CaCl_2_ data than with the MQ data.

## Discussion

### Advantages of the glass bead semi-hydroponic system

The glass bead semi-hydroponic plant growth system was effective in supporting the growth of healthy maize plants for exudate collection and phenotype analysis. The advantages of this system are multifold. First, the nutrient solution delivered to the plants during the entire experiment is sterile since the system uses a peristaltic pump to distribute sterile nutrient solution directly to the plants through autoclaved tubing. Moreover, the addition of a Teflon lid to cover the roots inside the glass tubes adds a barrier to protect the roots from environmental contamination. This system is set up inside a growth chamber to ensure optimal plant growth conditions, including controlled humidity, as fluctuations in humidity could impact growth and exudate profiles. In addition, this controlled environment allows for standardized collection procedures, including specific exudate collection times, to eliminate impacts on exudates by confounding factors such as time of day. A similar system used for tomato plants was successful in keeping the rhizosphere sterile [41]. Second, this system allows for continuous collection of root exudates with no disturbance to the roots, similar to rhizobox systems [42]. This non-destructive method is beneficial for studying root exudates as it reduces the risks of the introduction of artefacts from root damage and handling as occurs in soil or sand-based exudation sampling systems such as exudation traps [43, 44]. Plants transferred from soil to a hydroponic system for exudate collection need a recovery period of 3 days to reduce the impact of root damage and nutrient leaching on root exudation [45]. In our system, there is no recovery period required, and after root exudates are collected, roots can easily be harvested to scan and phenotype and to do further metabolomic or gene expression analysis. Finally, a semi-hydroponic system using glass beads mimics soil conditions better than hydroponic or aeroponic cultures. In addition to the lack of mechanical impedance in hydroponic or aeroponic systems, some studies have shown anatomical differences in the endodermis and exodermis differentiation of roots from hydroponic systems [46, 47]. In hydroponic systems without aeration, a lack of oxygen can cause hypoxic stress which results in increased suberization of the exodermis lamellae [46, 47]. Our system uses a combination of watering by intermittent dripping and flooding. The dripping allows the roots to be exposed to oxygen to avoid hypoxia and the flooding step avoids water deficit stress. Additionally, it is worth noting that the roots of the plants in our system formed root hairs, in contrast with the roots of maize grown in a hydroponic system (Additional file 3). Root hairs are important structures responsible for water and phosphorous uptake [48], as well as carbon exudation [49]. If the development of root hairs is compromised, it could result in the misinterpretation of the changes in root exudation profiles [41]. In conclusion, the design of our system provides for a semi-sterile root-growth environment with conditions that mimic the structure of soil and provides an easy way to continuously collect root exudates from undisturbed roots and to extract roots for phenotyping. The system can be scaled up to perform larger experiments.

### Compounds characterized in maize root exudates

Root exudate composition depends on many factors, foremost of which are the plant species and cultivar. Our study focused on the collection methods for the root exudates of maize, the composition of which has been studied before. However, comparisons of metabolites and their levels between studies are difficult because of the different growth conditions, developmental stages, genotypes used, and study designs of root exudates collection, i.e., collection solution and time points as summarized in Additional file 9 [30, 32, 50–53]. What is well established is that maize root exudates contain mostly sugars, organic acids, amino acids (including GABA), phenolics, fatty acids, sugar acids, and alcohols [29–31, 44, 51, 54–59]. A specific compound identified in root exudates of grasses is the benzoxazinoid DIMBOA. This compound has been found to have a critical role in recruiting plant beneficial microorganisms which affects plant growth [52, 60]. In our study, DIMBOA as well as a wide range of phytohormones with some (SA, JA, ABA. IAA) previously detected in root exudates of other plants, e.g., Arabidopsis [21], *Avena* [13], citrus [61] and tomato [62], were analyzed using targeted LC–MS/MS of maize root exudates. However, this is the first report of SA, JA, ABA, and IAA, as well as cZ, tZR, Me-IAA and JA-Ile detection in maize root exudates, collected with either water or CaCl_2_. Moreover, none of the conjugated forms of IAA (IAA-Asp, IAA-Ala, and IAA-Trp), or the gibberellins included in the targeted LC–MS/MS assay were detected in our samples. The recovery rates for the phytohormones assayed for but not detected in root exudates are listed in the Additional file 10. Their recovery was higher than 90%, except for OPDA which was at 21% after the MCX-SPE step. Here we showed that although the MCX-SPE step led to changes in the concentrations of some of the phytohormones (Table 1), it allowed for the removal of the CaCl_2_ and the recovery of hormones from the samples without introducing experimental variation. However, the levels of the hormones not detected in root exudates are either lower than the limits of detection of the LC–MS/MS assay (Additional file 11) or are not present in the root exudates.

In addition to phytohormones, we analyzed amino acids and sugars but did not analyze the organic acid composition since the MCX-SPE cartridge used was not compatible with their chemistry and the presence of CaCl_2_. The MCX-SPE step was essential for the CaCl_2_ collection method to eliminate interfering salts for the LC–MS/MS assay. Amino acids are secreted in root exudates and the concentration in exudates varies due to nitrogen availability in soil [63]. Here, 19 amino acids, including GABA, were detected in all samples collected with water or CaCl_2_, Glycine (Gly) and Cysteine (Cys) were not included in the method because of poor resolution and detection using the HILIC (Hydrophilic Interaction Liquid Chromatography) based LC–MS/MS method optimized for the study (Additional file 11). We found that the MCX-SPE step for exudates collected in CaCl_2_, was responsible for low recovery of some amino acids (Group 2 AA: Ala, Arg, Asn, Asp, Gln, Glu, Met, Ser, Thr) (Table 2b). Thus, we could only characterize a subset of amino acids (Group 1 AA: GABA, His, Leu, Ile, Lys, Phe, Pro, Tyr, Trp, Val) in the exudates. Similarly, Oburger et al. [32] was only able to detect 9 different amino acids when they compared maize root exudates collected in water or CaCl_2_ from plants grown in hydroponic solutions from a rhizobox/rhizotron [32]. The authors used a similar methodology that included an MCX-SPE step followed by HILIC-MS/MS analyses, and 7 out of 9 of the amino acids detected were common to the Group 1 AA defined in our study. It was previously reported that acidic charged side chains amino acids (Asp, Glu) and the basic charged side chain Arg were not recovered after MCX-SPE [40] which also concurs with our observations. The amino acids with the most affinity for the MCX-SPE cartridge are the ones with non-polar (Leu, Ile, Phe, Pro, Trp, Tyr Val, except for Met and Ala) and basic charged (His and Lys) side chains, which we classified in Group 1 AA and had the highest recovery rates (Table 2a). The amino acids with polar side chains (Asn, Gln, Ser, Thr) that would be expected to have the lowest recoveries, were detected in both our study and the study by Oburger et al. [32]. In contrast, when water was used for root exudate collection and no MCX-SPE step was required, all amino acids, except for Gly and Cys, and GABA were detected in maize root exudates. Therefore, this suggests that the use of the SPE with MCX cartridge is not appropriate for the study of all amino acids present in exudates. However, as we discuss below, collection of exudates with water poses other issues.

The last group of metabolites we detected and analyzed were the sugars using a derivatization methodology followed by GC–MS. The most abundant sugars detected were monosaccharides Ara, Fru, Gal, Glc, Man, Xyl and disaccharides Suc and Tre. Unlike the amino acids, the sugar abundance levels did not differ between root exudates collected in water or CaCl_2_. In addition, the presence of CaCl_2_ in the samples did not interfere with the assay.

This present study was able to characterize a large number of metabolites in maize root exudates, with some reported for the first time. Very recently, a large untargeted metabolomics study looking at profiling metabolites of root exudates in several plants including maize [56] reported over 8758 compounds with assigned empirical formulas, with 744 of them unique to maize. Because of the limitation of the acquired data based only on accurate mass, no metabolite identification can be confirmed, and it is not possible to know if any of the phytohormones identified in our study were detected in this untargeted study.

### Differences in metabolites levels between water and CaCl_2_ root exudation solutions

Our study showed that the solution used to collect root exudates impacts the concentrations of a subset of metabolites in exudates. Despite the significant losses of some amino acids from the use of the MCX-SPE cartridge, we showed that other metabolites included in this study are recovered at high and reproducible rates (Tables 1 and 2), thus enabling the comparison between water- and CaCl_2-_ collected exudates. The phytohormones levels measured were similar between the two solutions, however the overall concentration of amino acids was consistently higher for most genotypes in the root exudate collected in water compared to CaCl_2_ (Fig. 2b). Leakage of compounds due to damage in membrane integrity has been reported before [28, 32, 37, 38] and it is very likely that this is happening when water is used as a collection solution. Similar results were reported by Oburger et al. [32] with higher amino acid concentrations found in exudates collected in water compared to 0.5 M CaCl_2_. In contrast other studies have shown that the use of water for the collection of root exudates did not affect the concentrations of metabolites [34, 35, 63]. This may be due to differences in experimental design (growth conditions, collection time), and the analysis of only a small set of compounds. And as we show here, not all compounds seem to be affected by the nature of the solutions used for collection, as exudation of only amino acids and not phytohormones or sugars were affected. Although the mechanism of exudation of amino acids is not known, it is hypothesized that the concentration gradient between the root and the soil solution is driving the exudation of amino acids through passive transport [63]. The use of water for root exudation is likely responsible for exacerbating the diffusion of amino acids into the exudate through passive transport in combination with the loss of membrane integrity. While it has been reported that sugars secreted in high concentration in root exudates are transported through ion channels rather than just diffusion through the membrane, there is little to no information on how the phytohormones are transported [61].

### Metabolic and phenotypic correlations

In addition to the analysis of root exudates this study collected root phenotypes making this a novel approach to studying the natural variation in functional and morphological traits. We found that at least three replicate tubes with two plants per genotype were needed to allow for this analysis. Based on these preliminary observations, genotypes with the smaller root systems exude higher concentrations of amino acids in the rhizosphere as shown by the correlation analysis between exudates and measured traits (Fig. 6). The correlation between root development and amino acids synthesis and transport in roots, but not exudates has been previously studied [64] and it was suggested that the amino acid/nitrogen source and transport is modified based on the root development and the plant environment. The purpose and mechanisms of amino acids efflux from roots into the rhizosphere are part of an on-going debate [65]. The amino acid exudation from roots is the result of the balance between efflux and influx [66] that may be controlled by amino acid transporters, either uni- or bidirectional [67] which have mainly been studied in reproductive part of plants. Therefore, more research is needed to understand the mechanisms of efflux from roots and the impact exudates have on shaping the rhizosphere soil microbial communities.

## Conclusion

We designed and demonstrated the use of a new plant growth system that combines a glass bead-supported hydroponics and a semi-automated drip watering system for reproducible collection and analysis of root exudates and phenotypes. This growth system has several advantages over other systems, including that it provides a growth environment that mimics some aspects of the mechanical impedance roots experience in soil but in a semi-sterile and controlled environment. It also allows for the collection of root exudates without disturbing and damaging the roots. In testing this system with seven maize phenotypes, we found that we could reliably phenotype the roots and analyze subsets of amino acids, phytohormones and sugars using targeted metabolomic analysis. We showed that the choice of root exudate collection solution impacts the exudation of a subset of the analyzed metabolites. When exudates were collected in CaCl_2_, the MCX-SPE step was not compatible with the study of polar amino acids. Despite this, the recovery of the other amino acids as well as phytohormones and sugars was not affected by the CaCl_2_ collection methodology. We report here for the first time the detection of phytohormones in the root exudates of maize, and our comparison of the collection solutions confirmed that water is not suitable for the study of amino acids, as it seems to increase their exudation. Finally, the correlation analysis using the root exudate chemical characterization with phenotyping promises to provide a powerful tool to identify natural variation of populations by linking phenotype to genotype.

## Method

### Seed disinfection

Maize seeds were surface sterilized inside a fume hood with chlorine gas (Cl_2_) produced by mixing 3.3 mL of HCl with 100 mL of commercial bleach (The Clorox Co., Oakland, CA) in a beaker. The bleach solution was next placed in a vacuum desiccator to provide an airtight container where the selected seeds were placed in 24-well cell culture plates. The desiccator was sealed (no vacuum applied) and after an incubation of 24 h the procedure was repeated. Following the sterilization, the seeds were imbibed in sterile and aerated 1 mM CaCl_2_ for 24 h. Aeration was done by bubbling sterile filtered air into 250 mL glass containers. The seeds were transferred to Petri dishes containing sterile paper towels saturated with sterile 1 mM CaCl_2_ and kept in the dark at 30 °C for 5–7 days until germination.

### Plant growth system

A semi-sterile plant growth system was designed for capturing root exudates. Plants were grown in custom designed glass tubes (Adams and Chittenden Scientific Glass, Berkeley, CA) with 3 mm soda-lime beads as growth support (Fisher Scientific, Water Stern). The tubes were 30 cm in length, 51 mm outside diameter and 9.5 mm wall thickness, with a tapered bottom and glass tubing of 4 cm length and 1 cm outside diameter (Fig. 1a). Small indents were placed into the bottom of the taper to keep the glass beads in the tube. The glass tubing at the end of the glass tube was connected to a 5 cm length Viton^®^ tubing (OD (outside diameter) 1/8 in ID (inside diameter) 1/16 in) that could be clamped close with an Acetal clamp (0.45 OD) (Halkey-Roberts^®^, USA plastics) (Fig. 1b). To grow the plants, the glass tubes were filled with glass beads, leaving a 3 cm space at the top to allow planting of sterilized germinated seeds, the beads were covered with a Teflon lid (Fig. 1c) designed with two 1.5 cm diameter holes to allow the seeds to emerge and two 0.5 cm diagonal perforations for watering purposes (Fig. 1d). The two small perforations allow the insertion of a two Teflon tube connected to a Y-connector (attached to a 4 cm Teflon tubing sealed with Teflon tape) (Fig. 1e) into the Teflon lid (Fig. 1f). Prior to every use, all glass materials were rinsed ten times with tap water, soaked with soap overnight, rinsed ten times with diH_2_O, then soaked overnight in 0.5% nitric acid solution in MQ, followed by ten rinses with diH_2_O, and three rinses with MQ. The filled glass tubes and all connecting parts were autoclaved with the ends covered with aluminum foil.

### Planting

To maintain sterile conditions, planting was done inside a laminar flow hood. Before planting, the autoclaved glass tubes filled with beads were rinsed once with sterile full-strength Hoagland and Arnon’s nutrient solution (6.5 mM KNO_3_, 4 mM Ca(NO_3_)_2_·2H_2_O, 1 mM NH_4_H_2_PO_4_, 2 mM MgSO_4_·7H_2_O, 4.6 μM H_3_BO_3_, 0.5 μM MnCl_2_·4H_2_O, 0.2 μM ZnSO_4_·7H_2_O, 0.1 μM Na_2_MoO_4_, 0.5 μM CuSO_4_, 25 μM CaCl_2_, 71.4 μM Fe-EDTA (ethylenediaminetetraacetic acid) [68]. Next, the Teflon lid was removed to facilitate placement of two germinated seeds on top of the beads (Fig. 1g), after which the Teflon® lid was re-positioned such that the plumule was showing through the holes to allow the plants to grow (Fig. 1h). The Y-connector was inserted in the Teflon as shown (Fig. 1f). Next, the Acetal clamp on the Viton tubing at the bottom of the tube was closed and the glass tube was filled with sterile full-strength Hoagland and Arnon’s nutrient solution to a level just below the seeds and the Teflon lid was covered with a 2.5 cm layer of dry sterile glass beads, leaving 0.5 cm of space at the top (Fig. 1i). The glass tubes were then covered with aluminum foil, leaving room for the plant to emerge, and placed into the tube rack described below (Fig. 1j, 1). Once planted, the tube racks were kept in a growth chamber (light 16 h/26 °C, dark 8 h/18 °C, relative humidity ~ 60%). Immediately after the plants emerged, the aluminum foil was removed. The tube racks were 25 cm long, 5 cm thick-walled PVC (polyvinyl chloride) pipes, held together with a perforated sheet of PVC. These were elevated by 30 cm in a Plexiglas rack with three vertical panels holding the tube rack (Fig. 1j, 1).

### Irrigation system

To ensure consistent irrigation of all tubes we designed a semi-automated drip irrigation system composed of two main components; the first one was a line made of Vitube^®^ Flexible Tubing of Viton™ 1/4 in inside diameter (ID) × 3/8 in (outside diameter) OD × 1/16 in wall (New Age Industries, Inc) (Fig. 1j, 2) which takes the sterile Hoagland and Arnon’s nutrient solution from a 19 L glass Pyrex^®^ carboy (Fig. 1j, 3) using a peristaltic pump (PeriPump NE-9004, New Era Pump Systems, Inc) (Fig. 1j, 4). The second component was a 2 × 8 branched tubing manifold (Fig. 1k, 5) that delivers nutrient solution to each glass tube (Fig. 1k, 6). The Viton line was connected to the 2 × 8 branched tubing manifold through a 3-way connector (Ominift^®^ Teflon) (Fig. 1l, 7) using a male PEEK (polyetheretherketone) [barbed adapter 1/4–28 (Diba—Kinesis®, Inc)] (Fig. 1l, 8). The branched manifold was fabricated with fourteen compression fittings (Fig. 1l, 9) connected with twelve 3 cm Teflon^®^ tubing (OD 1/8 in ID ¼) (Fig. 1l, 10). Each of the sixteen output lines consisted of 30 cm Teflon^®^ tubing (OD 1/8 in ID ¼) (Fig. 1k, 6) connected to the Y-connectors that ultimately were inserted in the Teflon lids inside the glass tube, to irrigate each tube individually (Fig. 1k, 6). All the tubing of the watering system was autoclaved before each use. To irrigate the plants sterile full-strength Hoagland and Arnon’s nutrient solution was made by diluting concentrated stocks solution into MQ filtered with the Millipak^®^ Express 40–0.22 µm (Merck KGaA, Darmstadt, Germany) in autoclaved carboys. The carboys were covered with black 3 mm plastic bags to keep solutions in dark (Fig. 1j, 3).

### Irrigation protocol

After planting, the tubes were kept filled with nutrient solution for 4–5 days until all the plants had emerged, after which time the irrigation described above was set and started. The irrigation scheme was done in a stepwise procedure to adapt to the needs of the growing plants as shown in Table 3. This was the optimal watering schedule for inbred maize that may need to be further optimized for other plant species. The watering system allowed for intermittent drip irrigation and flooding of the glass tubes, in a similar process used in a “flood and drain” hydroponic system. The flooding cycle prevented the seedlings from drying out and reduced the need to constantly replenish the carboys with nutrient solution. When flooded, the nutrient solution was in contact with the roots and not with the seeds. When intermittently irrigating, we chose not to recirculate the nutrient solution to keep the input solution sterile throughout the growth of the plants, and to keep the solution running and drained to ensure the roots were never exposed to anoxic conditions.

**Table 3:** Irrigation protocol

Days after planting	Time that plants were drip irrigated hours/schedule	Time plants were flooded hours/schedule	Volume supplied—pause (total volume per hour)
4 or 5	4 h/2–6 p.m	18 h/6–12 p.m. next day	30 mL–30 s (3600 mL/h)
6	4 h/12–4 p.m 2 h/6–8 p.m	2 h/4–6 p.m 16 h/8–12 p.m. next day	35 mL–40 s (3150 mL/h)
7	4 h/12–4 p.m 4 h/6–8 p.m	2 h/4–6 p.m 16 h/8 –12 p.m. next day	35 mL–50 s (2520 mL/h)
8	4 h/12–4 p.m 14 h/8 p.m.–10 a.m. next day	4 h/4–8 p.m	35 mL–60 s (2100 mL/h)
9	20 h/2 p.m.–10 a.m. next day	4 h/10 a.m.–2 p.m	35 mL–70 s (1800 mL/h)
10	20 h/2 p.m.–10 a.m. next day	4 h/10 a.m.–2 p.m	35 mL–70 s (1800 mL/h)
11	20 h/2 p.m.–10 a.m. next day	4 h/10 a.m.–2 p.m	35 mL–70 s (1800 mL/h)
12	20 h/2 p.m.–10 a.m. next day	4 h/10 a.m.–2 p.m	35 mL–70 s (1800 mL/h)
13	18.5 h/2 p.m.–8:30 a.m. next day	4 h/10 a.m.–2 p.m	40 mL–70 s (2057 mL/h)
14	Exudates were collected with 1 mM CaCl_2_ at 10:30 a.m		
	1.5 h/10:30 a.m.–12:00 p.m 18.5 h / 2 p.m.—8:30 a.m. next day	2 h/12–2 p.m	40 mL/70 s (2057 mL/h)
15	Exudates were collected with MQ at 10:30 a.m Roots weighted and scanned afterwards		

The first 2 days after the watering system was set up, the total volume supplied was greater than the consecutive days because the roots were small, and the plants were very susceptible to desiccation. From the 6th day after planting, a reduction of the total volume supplied per hour was made, making sure that the plants were not water-stressed. As the plants got bigger, the total volume supplied was increased from day 13th after planting. We determined the rate and volume needed by trial and error in preliminary experiments. A constant nutrient flow rate (350 mL/s) was used to supply the nutrient solution, only changing the volume supplied and the length of time in each irrigation cycle.

### Experimental design and collection of exudates

We designed an experiment to collect and analyze root exudates from seven genotypes of the Buckler-Goodman diversity panel [69]. We collected exudates with 1 mM calcium chloride (CaCl_2_) and ultrapure water (MQ). To compare results, root exudates were collected from the same plants on two successive days. On the 14th day after planting, exudates were collected with autoclaved 1 mM CaCl_2_, and with autoclaved MQ on the 15th day after planting. Three to four replicate tubes were planted with two plants each of the following genotypes: Ames 12734, Ames 20140, Ames 20190, Ames 27171, Cize 7, NSL 22629, and PI 587154. The root exudates were collected in the growth chamber, always at the same time of the day to avoid any diurnal effects, at 10:30 a.m., after the tubes had been flooded for two hours. All processes were done without disturbance or removal of the plants from the glass tubes. Flooding was done by first draining tubes completely and then the Viton tubing was closed using the Acetal clamp. First, the glass tubes were filled twice with the collection solution, soaked for 1 min, and drained. The tubes were then filled with 150 mL of the collection solution, a volume sufficient to allow for total submergence of the roots without contact with the seed, to avoid collecting seed exudates as has been pointed out before [70].

After a 2-h incubation period the solution was drained into 250 ml glass jars (VWR International, I-CHEM), and immediately placed on dry ice to transport them to a − 80 °C freezer where they were kept for 24 h. Next, the samples were freeze dried in a 12 L Bulk tray Dryer (Labconco Corporation, Kansas City, MO), with a − 50 °C condenser and 0.02 mbar chamber pressure. The top of the jar was covered by a piece of aluminum foil, a plastic lid, and filter paper (Whatman, 55 mm ∅, Cat No.: 1001-055). The aluminum foil and the lid both had a perforation of 2 cm diameter in the center to allow the lyophilization to proceed.

### Sample preparation for LC–MS/MS and GC–MS analyses

Freeze dried samples from exudates collected with both 1 mM CaCl_2_ and MQ, were resuspended in 8 mL of precooled 2% formic acid in MQ. Each sample was then split into two 4 mL samples and transferred to 15 mL falcon tubes. Half (4 mL) sample was used for phytohormones, DIMBOA and amino acids (including GABA) analysis using LC–MS/MS and was spiked with 5 μL of an internal standard (D5IAA, D2JA, D4SA, D6ABA, D5tZR and D5tZ at 0.83 µM; D2GA1 at 5 µM; NVa at 46.7 µM). The other 4 mL was used for sugars analysis using GC–MS and was spiked with 10 μL pinitol at 1 mM as an internal standard. All the samples were stored at − 80 °C until analysis.

The samples collected with 1 mM CaCl_2_ to be analyzed by LC–MS/MS, underwent an extra clean-up step to remove salts by using MCX-SPE cartridges (Oasis MCX 1 cc Vac Cartridge, 30 mg sorbent, 30 µm, Waters) to reduce interference with the LC–MS/MS. All the steps were done using a vacuum manifold. The MCX-SPE cartridges were first conditioned by running through 2 × 1 mL of 100% methanol, followed by an equilibration step of 2 × 1 mL MQ-water. The 4 mL samples were next loaded on the cartridge, followed by three washes with 500 µL of 2% formic acid in MQ. The compounds were eluted from the cartridges in two steps, first with 2 × 250 µL 100% methanol and second with 2 × 250 µL 5% ammonium hydroxide/95% methanol. The elutes were combined in the same tube and stored at − 80 °C until ready to be dried down in the SpeedVac. Samples collect in MQ did not require clean up by MCX-SPE cartridges.

### Targeted LC–MS/MS analysis of phytohormones, DIMBOA and amino acids

Phytohormones, DIMBOA and amino acid analysis of the root exudates was done by LC–MS/MS using Multiple Reaction Monitoring (MRM) scan mode. First, the samples from the MCX-SPE clean-up step (collected with 1 mM CaCl_2_) and those collected with MQ were dried down in a SpeedVac and resuspended in 100 µL of 30% methanol. For the phytohormones analysis, a 15 µL aliquot was diluted two times with MQ and transferred into the HPLC vials, ABA, SA, JA, JA-Ile, OPDA, IAA, IAA-Asp, IAA-Ala, IAA-Trp, Methyl IAA, gibberellins (GAs) 1,3,4,8,9,12,19,20, and 53, c-and t-zeatin, t-zeatin riboside, strigol and, DIMBOA were separated using a ZORBAX Eclipse Plus C18 column (2.1 × 100 mm, Agilent) running at a flow rate of 0.45 mL/min. The gradient of the mobile phases A (0.1% formic acid in water) and B (0.1% formic acid/90% acetonitrile) was as follow: 5% B for 1 in 4 min, to 100% B in 2 min, hold at 100% B for 3 min, to 5% B in 0.5 min. The column compartment was set at 40 °C.

For the amino acid analysis (including GABA), a 15 µL aliquot was dried down and resuspended in 70 µL 60% acetonitrile. The method allowed analysis of 18 amino acids: all amino acids but cysteine and glycine. Samples were separated on a XBridge Amide 3.5 µm (4.6 × 100 mm, Waters) at a flow rate of at 0.8 mL/min. The gradient of the mobile phases A (0.1% formic acid in acetonitrile) and B (0.1% formic acid in water) was as follows: 10–70% B in 7.4 min, hold at 70% for 3 min, back down to 10% B in 0.3 min. The column compartment was set at 45 °C.

The Shimadzu LC system used was interfaced with a Sciex QTRAP 6500 + mass spectrometer equipped with a TurboIonSpray (TIS) electrospray ion source. Analyst software (version 1.6.3) was used to control sample acquisition and data analysis. The QTRAP 6500 + mass spectrometer was tuned and calibrated according to the manufacturer’s recommendations. The mass spectrometer was operated with the IonDrive Turbo V electrospray ionization (ESI) source in positive and negative ion modes for the hormones and only in positive ion mode for the amino acids. The ESI source operation parameters were as follows: source temperature at 500 °C; ion spray voltage at 5500 for positive and − 4500 for negative ion mode; ion source gas 1 at 50; ion source gas 2 at 50; curtain gas at 20 psi; collision gas at medium. The hormones and amino acids were detected using MRM transitions that were optimized using standards. The MRM transition (Q1–Q3), compound settings (DP and CE), as well as the lower limit of quantification (LLOQ), the relative standard deviation (%RSD) and calibration range for each compound are provided in Additional file 11. For quantification, an external standard curve was prepared using a series of standard samples containing different concentrations of unlabeled compounds and fixed concentrations of the internal standards. Because there is no internal standard commercially available for DIMBOA, the average of all the hormones’ internal standards was used for normalization of the experimental variation.

### GC–MS single ion monitoring (SIM) analysis of sugars

For the GC–MS analysis of sugars, half of the 4 mL aliquoted sample was used (2 mL). First, the samples were dried down in a SpeedVac and then resuspended in 20 mg/mL methoxyamine hydrochloride reagent prepared in pure pyridine and incubated for 2 h at 37 °C on a platform shaker at 1000 rpm. Next, for derivatization, the MSTFA + 1% TMCS derivatization (ThermoFisher Scientific) was added to each sample, incubated for 30 min at 37 °C on a platform shaker at 1000 rpm followed by a centrifugation for 10 min at 16,000 g prior to transferring the mixture to GC vials for injection into GC–MS. The GC–MS analysis was carried out with an Agilent GC (Model 7890B) using electron impact (EI) and MS Quadrupole (Model 5977A) (Agilent Technologies, Santa Clara, CA, USA). The liquid injection was done using a PAL System RSI 85 (PAL, Lake Elmo, MN, USA). The injector temperature was 230 °C; the MS transfer line was 300 °C. Sugars were separated on a HP-5MS 30 m, 0.25 mm, 0.25 μm capillary column (Agilent Technologies), at constant flow 1.5 ml × min^−1^ of helium as a carrier gas. One microliter of derivatized sample was injected into the injector operating in splitless mode. The temperature of the column was initially set to 80 °C and increased at a rate of 15 °C × min^−1^ to 175 °C, followed by an increased at 5 °C × min^−1^ to 220 °C, and a final ramping to 320 °C at 25 °C × min^−1^. A SIM scan method using selected ions (Additional file 11) was used to analyze the sugars (xylose, arabinose, fructose, glucose, mannose, galactose, lactose, sucrose, trehalose and raffinose). The data was acquired at a scan speed of 3.125 μ/s with a dwell time of 40 ms for each ion selected. The generated data was analyzed with Agilent Mass Hunter Quantitative Analysis. For quantification, an external standard curve was prepared using a series of standard samples containing different concentrations of sugars and fixed concentration of the internal standard.

### Preparation of standards mixture to evaluate the experimental recovery of metabolites using MCX-SPE for LC–MS/MS analysis

We designed an experiment to determine the potential impacts that the MCX-SPE clean-up step had on metabolite recovery for exudates collected in CaCl_2_. A mixture of known concentrations of phytohormones and amino acids (Additional file 12) was prepared and spiked with the same internal standards as described above. This mixture was split in two, one half was adjusted to contain the same CaCl_2_ concentration used for exudate collection, and the other half just suspended in water. Both samples were processed through an MCX-SPE step and analyzed by LC–MS/MS as described above. The experimental variation from the MCX-SPE step was determined and the percentage recovery for each compound was calculated based on the peak area from the control sample, i.e., the same mixture of standards at known concentrations prepared in MQ. Five technical replicates of each condition (SPE-CaCl_2_, SPE-MQ, MQ-no SPE) were run and analyzed.

### Preparation of CaCl_2_ samples from the exudate samples collected with MQ

To determine the effect of the CaCl_2_ compared to water on root exudation and the analysis of root exudates, the root exudates from the same plants were also collected using MQ and analyzed by LC–MS/MS and GC–MS as described above. To compare the real effect of CaCl_2_ versus the effect of using the MCX-SPE with CaCl_2_, the water samples were split into four 2 mL samples, two of which were supplemented with concentrated CaCl_2_ to be directly comparable with the root exudates samples collected in 1 mM CaCl_2_. One 2 mL aliquot with CaCl_2_ was readjusted to 4 mL before MCX-SPE and analyzed by LC–MS/MS, the other one was analyzed by CG-MS as described previously. The water samples without CaCl_2_ were dried down and analyzed by LC–MS/MS and GC–MS as described above. Each sample was spiked with the same concentration of internal standard as previously described.

### Statistical analysis of the metabolomics results

To analyze the LC–MS/MS and GC–MS results, the concentration of each compound was normalized by the fresh root weight (FRW) and all zeros transformed to 0.0001. T-tests and correlation analysis and the graph building were performed using R v4.0.2 [71] through RStudio v1.2.5001 [72] and using the dplyr v 1.0.7, ggplot2 v 3.3.2, ggpubr 0.4.0, ggsignif v 0.6.3, tidyverse 1.3.1, corrplot v 0.90. The Tukey–Kramer HSD test was performed using JMP [73]. To perform the statistical analysis the data were transformed to Log2, and the graphs were drawn using the raw data to show the actual concentration. Aesthetic modifications to the graphs were made using Inkscape [74].

### Root scanning and phenotypic analysis

After the root exudates were collected, the glass beads were gently removed from the tubes, to prevent damaging the roots. Next, the roots were cut at the mesocotyl to separate roots from shoots. Roots and shoots were weighed separately, and roots scanned individually. Each root was placed on the scanner screen (Epson Perfection V800 Photo scanner, Epson America, Inc.) and moistened with 1 mM CaCl_2_ and manually spread to separate the different types of roots. To retrieve the images, the SilverFast SE software was used (LaserSoft Imaging, Inc.), and flipped and inverted using Adobe Photoshop. The pictures were submitted to the website of DIRT software for root trait analyses [39]. The masking threshold was set to 10.0 to remove the background noise and all the default settings. The shoots were photographed for image analysis and for a record of plant health. Only the relevant data retrieved by DIRT was shown in the results.

## Supplementary Information

**Additional file 1: Figure S1.** Scanned roots of seven maize genotypes grown in the glass bead-semi hydroponic system. Three images representative of each genotype are shown. The roots belong to plants grown 15 days after planting and after exudates were collected.

**Additional file 2: Figure S2.** Images of shoots of seven genotypes grown in the glass bead-semi hydroponic system. Three representative pictures of each genotype are shown. The plants shown were grown for 15 days after planting. Red square = 1cm^2^.

**Additional file 3: Figure S3.** Comparison of root morphology between maize plants growing in the glass bead semi-hydroponic system and hydroponics. **a** Root morphology of four genotypes of corn grown in glass bead semi-hydroponic system and hydroponics. Different types of roots are explained in the images of the genotype PI 587154 as an example. **b** Two corn genotypes grown in different substrates: glass bead semi-hydroponic, hydroponics, sand, and soil. **c** Close-up to selected images to illustrate the presence of root hairs in the plants growing in the glass bead semi-hydroponic system but not when the plants are grown using hydroponics.

**Additional file 4: Figure S4.** Differences in the concentration of phytohormones and DIMBOA detected in exudates collected with 1 mM CaCl_2_ and MQ in seven genotypes. Analysis of the effect of CaCl_2_ and MCX-SPE clean-up in the recovery of exudates. Different compounds are shown in two panels. **a** ABA (abscisic acid), cZ (cis-Zeatin), DIMBOA (2,4-dihydroxy-7-methoxy-1,4-benzoxazin-3-one), IAA (indole-3-acetic acid). **b** JA (jasmonic acid), JA-Ile (jasmonic acid-isoleucine, Me-lIAA (methyl- indole-3-acetic acid), SA (salicylic acid), tZR (trans-zeatin riboside). FRW (Fresh root weight). T test, each pair, only significant differences shown *p ≤ 0.05, **p ≤ 0.01. Cize 7, Ames 12734, Ames 20140, Ames 27171, NSL 22629 n = 3; Ames 20190, PI 57154 n = 4. Boxplot: Box, interquartile range (IQR); line inside the box, median; end of the box, upper (Q3) and lower (Q1) quartiles; dots beyond the extreme lines show potential outliers.

**Additional file 5: Figure S5.** Differences in the concentration of the group 1 of amino acids detected in exudates collected with 1 mM CaCl_2_ and MQ in seven genotypes. Analysis of the effect of CaCl_2_ and MCX-SPE clean-up in the recovery of exudates. Different compounds are shown in two panels. **a** GABA (Gamma aminobutyric acid), His (histidine), Ile (isoleucine), Leu (leucine), Lys (lysine). **b** Phe (phenylalanine), Pro (proline), Trp (tryptophan), Tyr (tyrosine), Val (valine). FRW (Fresh root weight). T test, each pair, only significant differences shown *p ≤ 0.05, **p ≤ 0.01. Cize 7, Ames 12734, Ames 20140, Ames 27171, NSL 22629 n = 3; Ames 20190, PI 57154 n = 4. Boxplot: Box, interquartile range (IQR); line inside the box, median; end of the box, upper (Q3) and lower (Q1) quartiles; dots beyond the extreme lines show potential outliers.

**Additional file 6: Figure S6.** Differences in the concentration of the group 2 of amino acids detected in exudates collected with 1 mM CaCl_2_ and MQ in seven genotypes. Analysis of the effect of CaCl_2_ and MCX-SPE clean-up in the recovery of exudates. Different compounds are shown in two panels. **a** Ala (alanine), Arg (arginine), Asn (asparagine), Asp (aspartic acid). **b** Gln (glutamine), Glu (glutamic acid), Met (methionine), Ser (serine), Thr (threonine). FRW (Fresh root weight). T test, each pair, only significant differences shown *p ≤ 0.05, **p ≤ 0.01. Cize 7, Ames 12734, Ames 20140, Ames 27171, NSL 22629 n = 3; Ames 20190, PI 57154 n = 4. Boxplot: Box, interquartile range (IQR); line inside the box, median; end of the box, upper (Q3) and lower (Q1) quartiles; dots beyond the extreme lines show potential outliers.

**Additional file 7: Figure S7.** Differences in the concentration of sugars detected in exudates collected with 1 mM CaCl_2_ and MQ among genotypes. **a** Sugar content in four genotypes. **b** Sugar content in the root exudates of genotype NSL 22629 (n = 3), effect of CaCl_2_ on the recovery of sugars. **c** Sugar content in the root exudates of genotype Ames 20140 (n = 3), effect of CaCl_2_ on the recovery of sugars. Ara (arabinose), Fru (fructose), Gal (galactose), Glc (glucose), Man (mannose), Suc (sucrose), Tre (trehalose), Xyl (xylose). FRW (Fresh root weight). T test, each pair, *p ≤ 0.05, only significant differences shown. Boxplot: Box, interquartile range (IQR); line inside the box, median; end of the box, upper (Q3) and lower (Q1) quartiles; dots beyond the extreme lines show potential outliers.

**Additional file 8: Figure S8.** Genotypic differences in the concentration of phytohormones and amino acids detected in exudates collected with MQ and 1 mM CaCl_2_. **a** Phytohormones detected in calcium chloride. **b** Phytohormones detected in MQ. **c** Amino acids detected in calcium chloride. **d** Amino acids detected in MQ. Statistical differences detected by All pairs Tukey–Kramer HSD, α = 0.05. Measurements with different letters within each graph are significantly different. Boxplot: Box, interquartile range (IQR); line inside the box, median; end of the box, upper (Q3) and lower (Q1) quartiles; dots beyond the extreme lines show potential outliers.

**Additional file 9: Table S1.** Comparison of the levels of amino acids, sugars and DIMBOA detected in this paper with those detected in previous studies.

**Additional file 10: Table S2.** Phytohormones not detected in root exudates. Percentage recovery before and after normalization. Percentages of the phytohormones after SPE. Recoveries were calculated before and after normalization using the internal standards D2GA1 for GAs 1, 3, 4, 8, 9, 12, 19, 20 and 53, D5IAA for IAA-Ala, IAA-Asp and IAA-Trp, D2JA for OPDA, D5tZ for tZ. Coefficient of variation (CV) are also included.

**Additional file 11: Table S3.** List of MRM transitions optimized for the hormones (Table 1) and amino acid (Table 2) with declustering potential (DP) and collision energy (CE) values, as well as the lower limits of quantification (LLOQ) in µM, the relative standard deviation (%RSD) and the calibration range. (Table 3) List of SIMs used for the sugars, as well as the lower limits of quantification (LLOQ) in µM, the relative standard deviation (%RSD), and the calibration range.

**Additional file 12: Table S4.** List of compounds with their concentrations used to make the standard mixture for the SPE test.

## Data Availability

The datasets used and/or analyzed during the current study are available from the corresponding author on reasonable request.

## Abbreviations

NaN_3_: Sodium azide
MES: 2-(N-morpholino) ethanesulfonic acid
KOH: Potassium hydroxide
diH_2_O: Deionized water
HPLC: High-performance liquid chromatography
CaCl_2_: Calcium chloride
MQ: Ultra-pure water obtained with a Milli-Q^®^ system
LC–MS/MS: Liquid chromatography—Tandem mass spectrometry
GC–MS: Gas chromatography—mass spectrometry
ABA: Abscisic acid
cZ: Cis-zeatin
DIMBOA: 2,4-Dihydroxy-7-methoxy-1,4-benzoxazin-3-one
IAA: Indole-3-acetic acid
JA: Jasmonic acid
JA-Ile: Jasmonyl-isoleucine
Me-IAA: Methyl indole-3-acetic acid
OPDA: Cis-12-oxo-phytodienoic acid
tZR: Trans-zeatin riboside
SA: Salicylic acid
D5tZ: D5-trans-zeatin
D4SA: D4-salicylic acid
D6ABA: D6-abscicic acid
D5tZR: D5-trans-zeatin riboside
D5IAA: D5-indole-3-acetic acid
D2JA: D2-jasmonic acid
GAs: Gibberellins
NVa: Norvaline
His: Histidine
Ile: Isoleucine
Leu: Leucine
Lys: Lysine
Met: Methionine
Phe: Phenylalanine
Pro: Proline
Trp: Tryptophan
Tyr: Tyrosine
Val: Valine
GABA: Gamma aminobutyric acid
Ala: Alanine
Arg: Arginine
Asn: Asparagine
Asp: Aspartic acid
Gln: Glutamine
Glu: Glutamic acid
Ser: Serine
Thr: Threonine
Ara: Arabinose
Fru: Fructose
Gal: Galactose
Glc: Glucose
Man: Mannose
Suc: Sucrose
Tre: Trehalose
Xyl: Xylose
FRW: Fresh root weight
DIRT: Digital imaging of root traits
SPE: Solid phase extraction
MCX: Mixed mode strong cation-exchange
CV: Coefficient of variation
IS: Internal standard
Cl_2_: Chlorine
OD: Outside diameter
ID: Inside diameter
EDTA: Ethylenediaminetetraacetic acid
PVC: Polyvinyl chloride
PEEK: Polyetheretherketone
SIM: Single ion monitoring
ESI: Electrospray ionization
MRM: Multiple reaction monitoring
Q1/Q3: Quadrupole 1/3
DP: Declustering potential
CE: Collision energy
LLOQ: Lower limit of quantification
RSD: Relative standard deviation
