# Supplementary material for: A glass bead semi-hydroponic system for intact maize root exudate analysis and phenotyping
Source: Plant Methods. 2022 Mar 5;18:25. doi: 10.1186/s13007-022-00856-4 (PMC8897885; doi:10.1186/s13007-022-00856-4)

Ames 12734
Root structure, scanned images

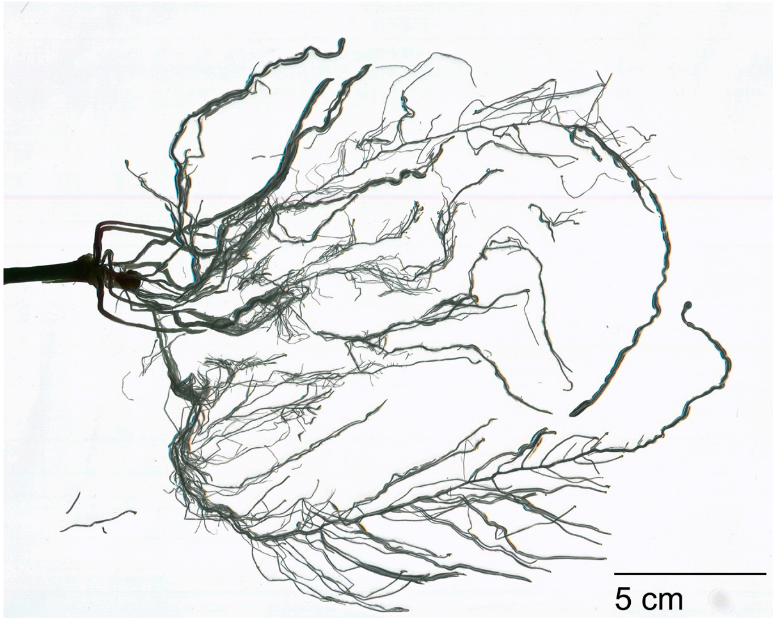

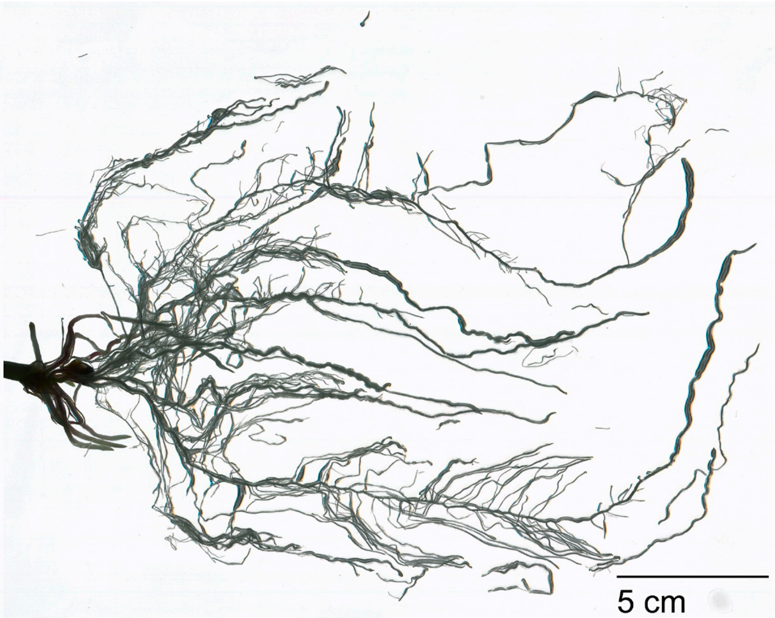

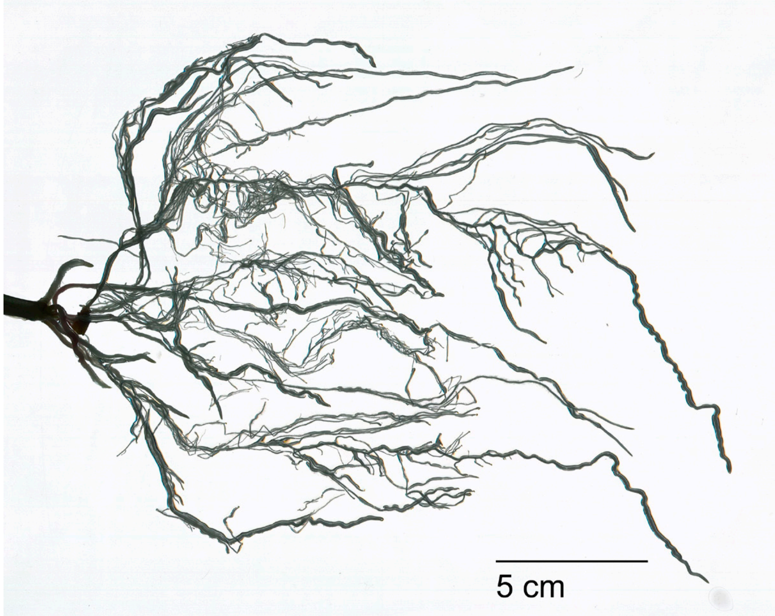

Ames 20140
Root structure, scanned images

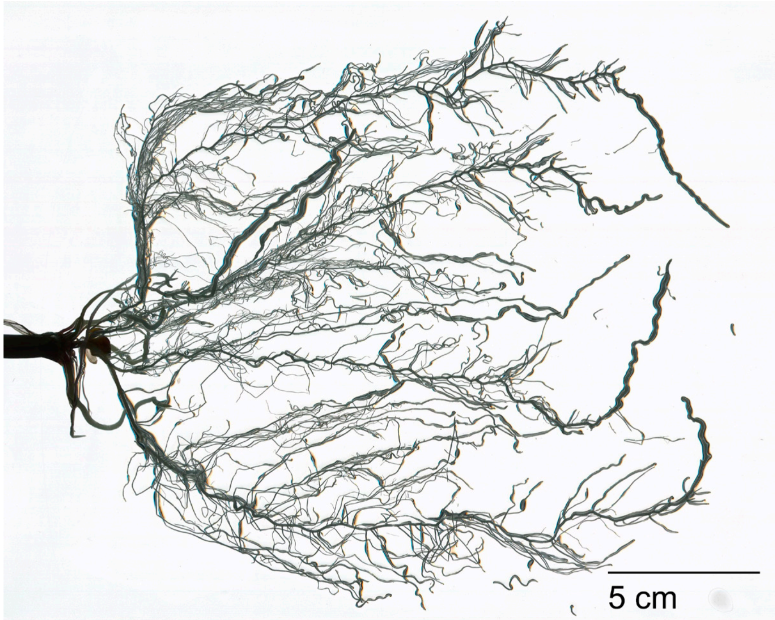

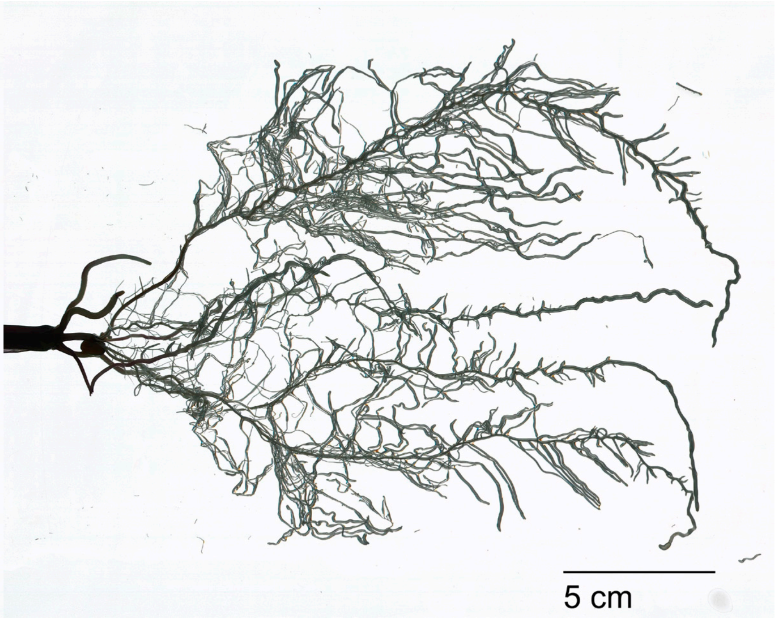

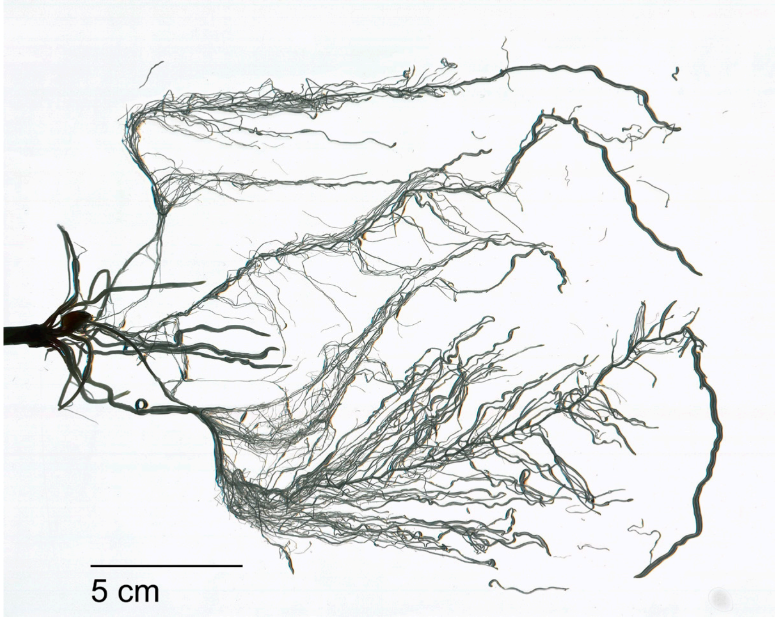

Ames 20190

Root structure, scanned images

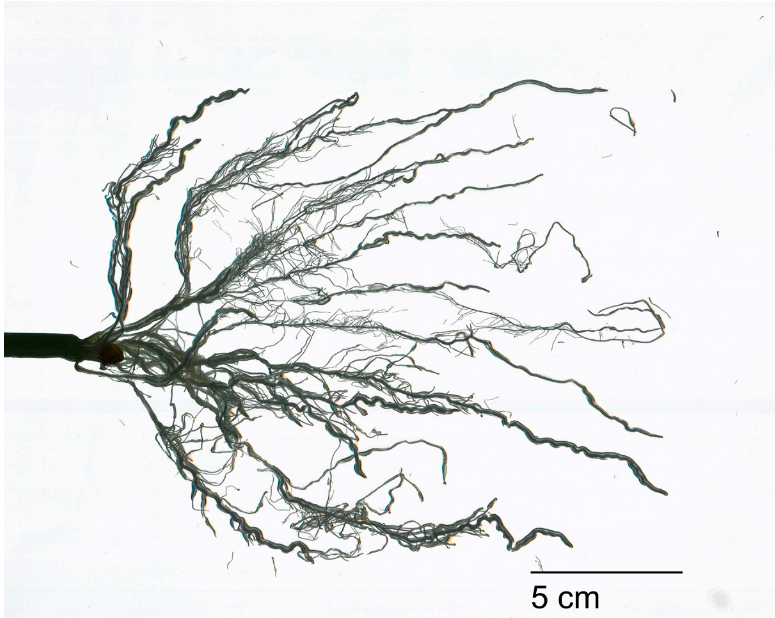

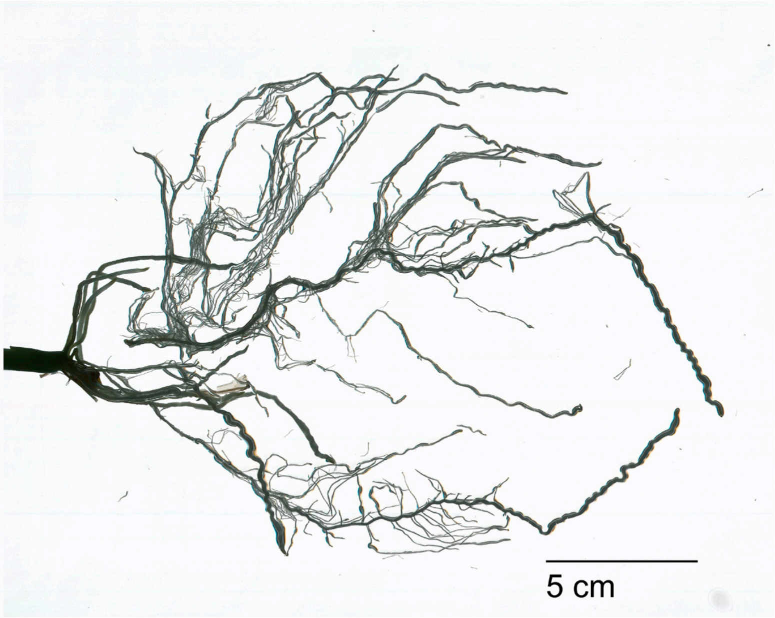

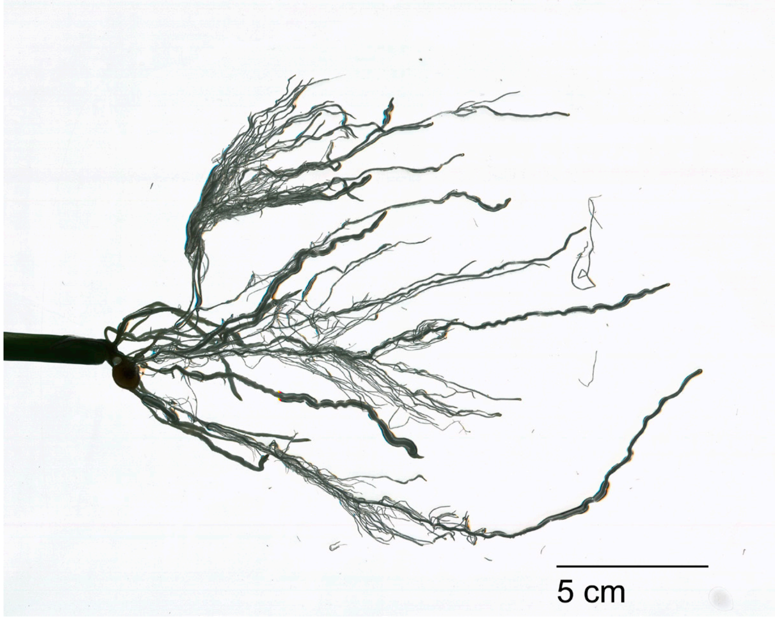

Ames 27171
Root structure, scanned images

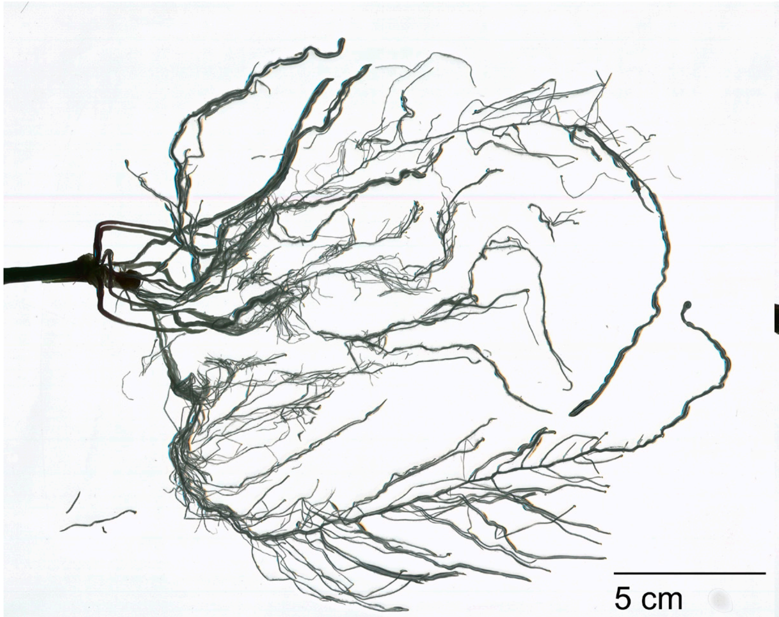

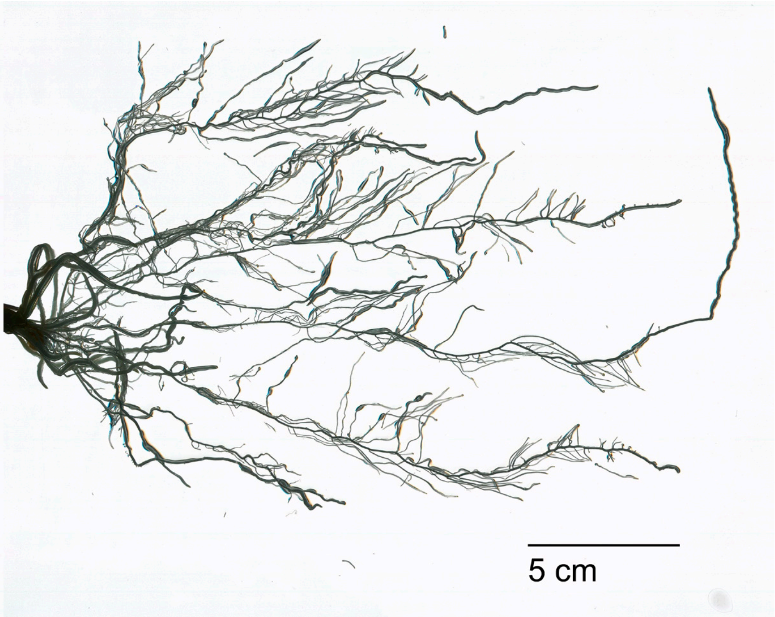

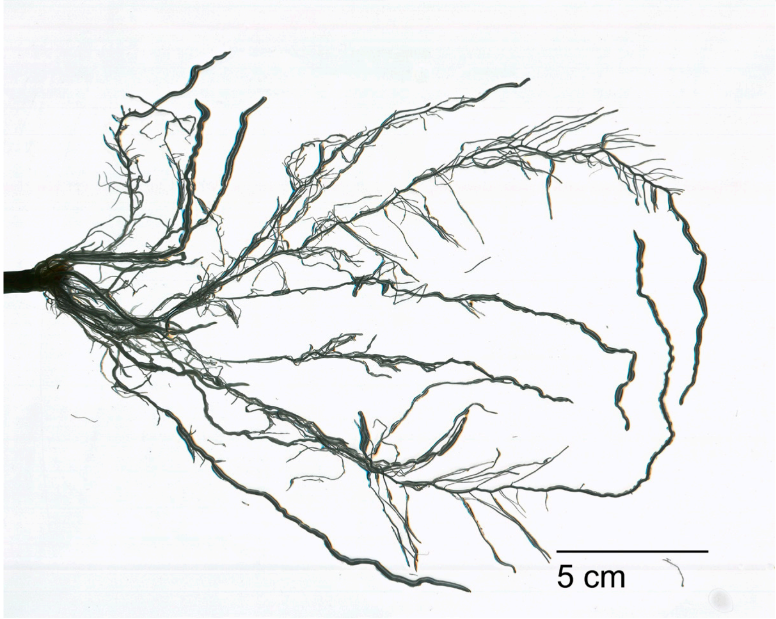

Cize 7
Root structure, scanned images

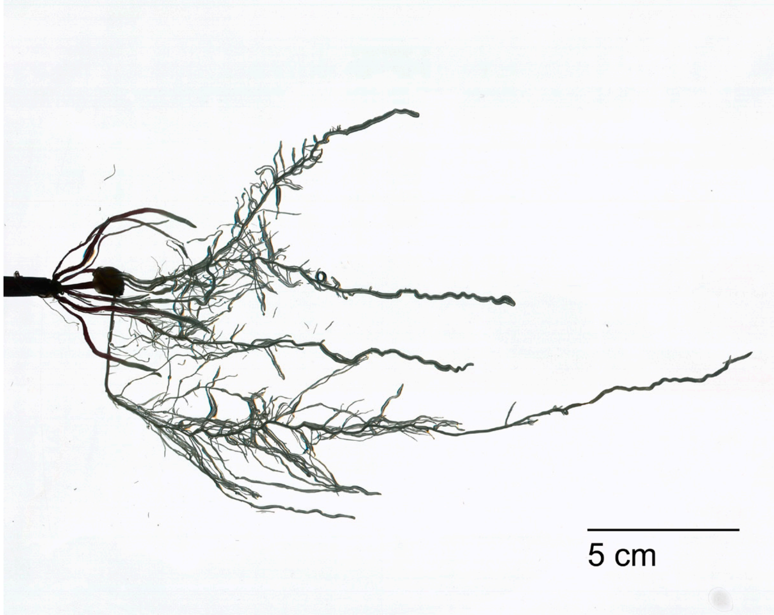

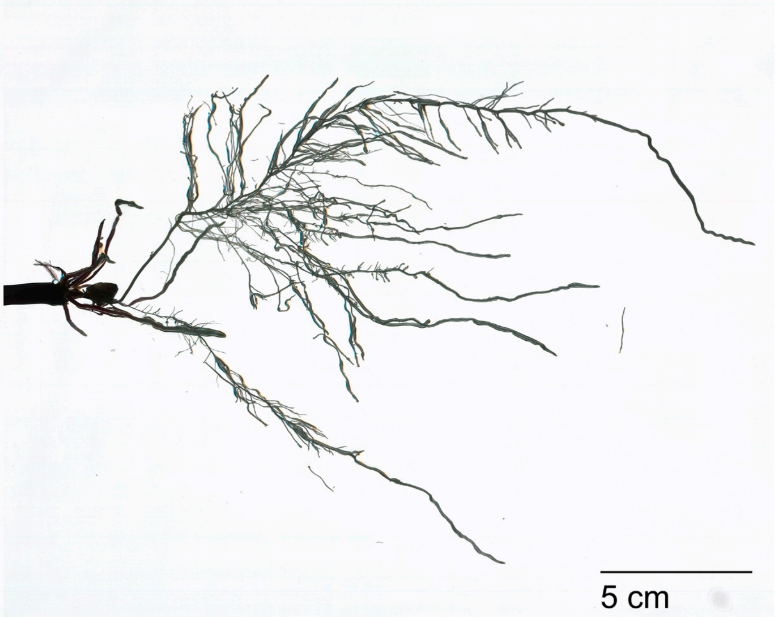

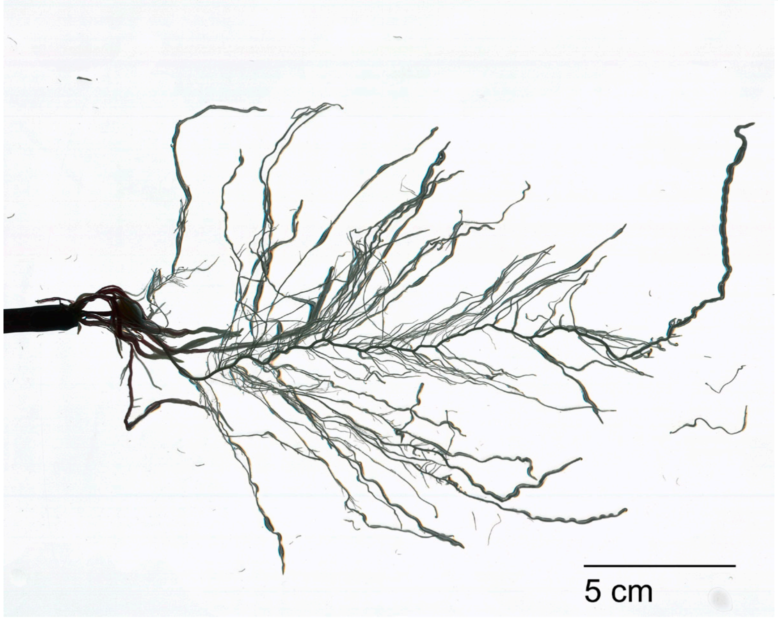

NSL 22629
Root structure, scanned images

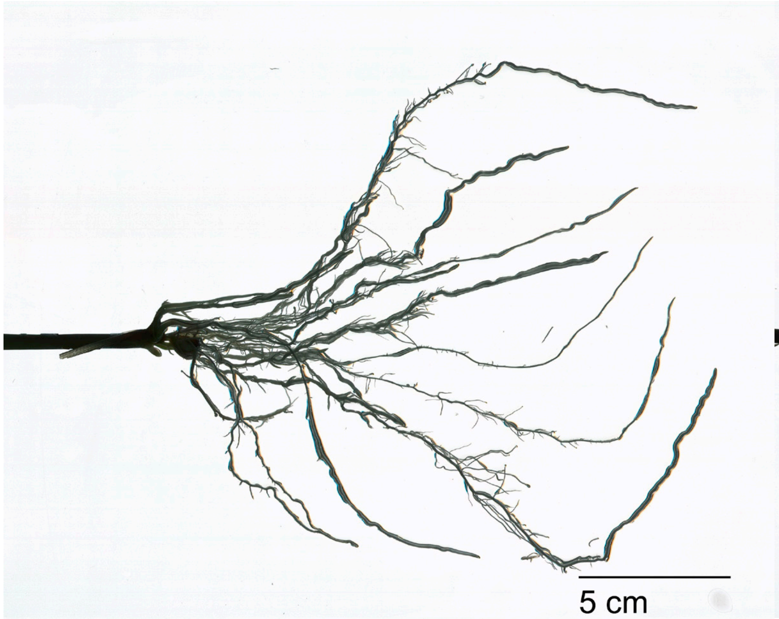

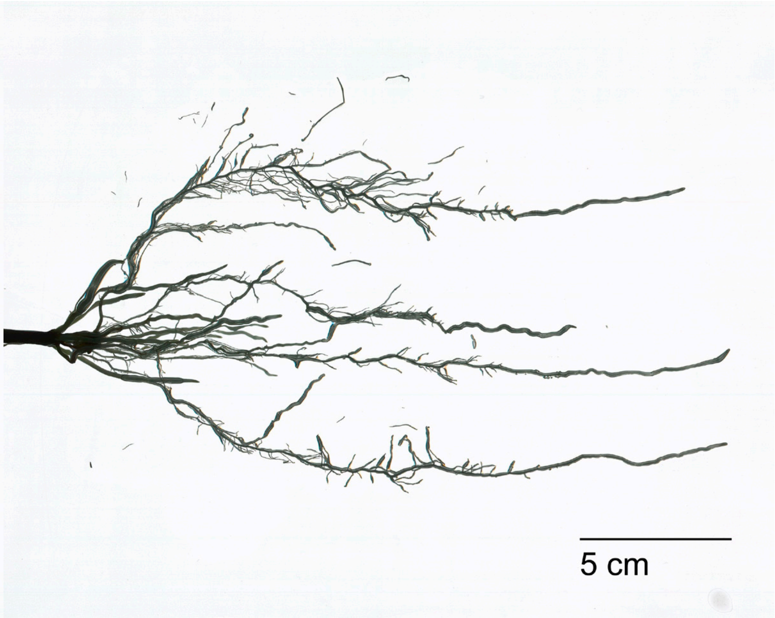

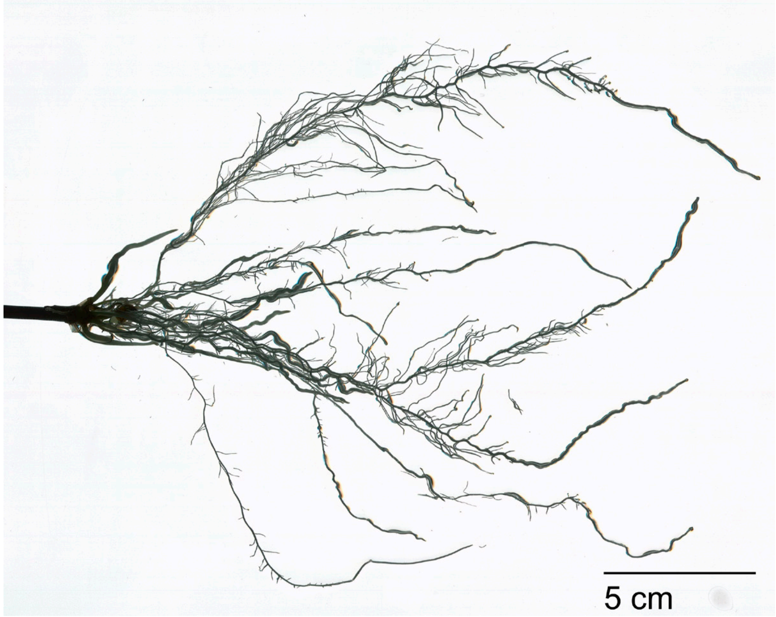

PI 587154
Root structure, scanned images

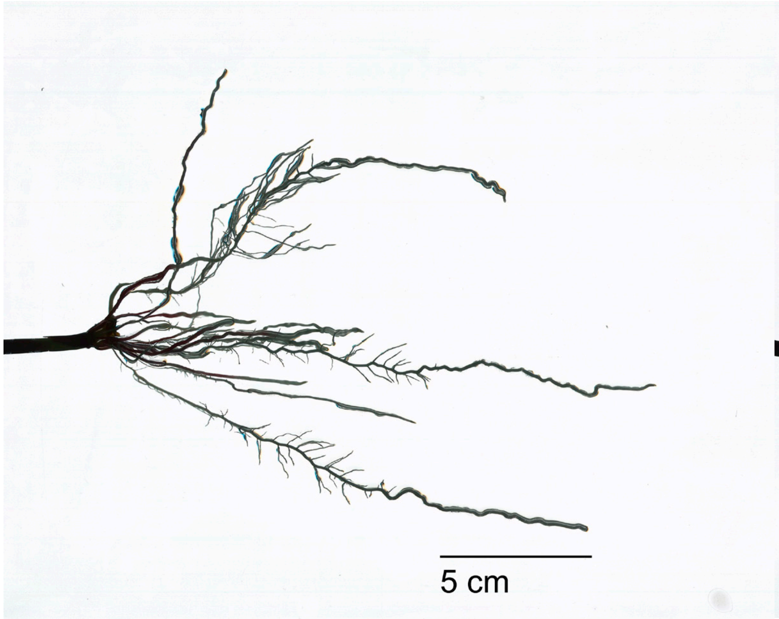

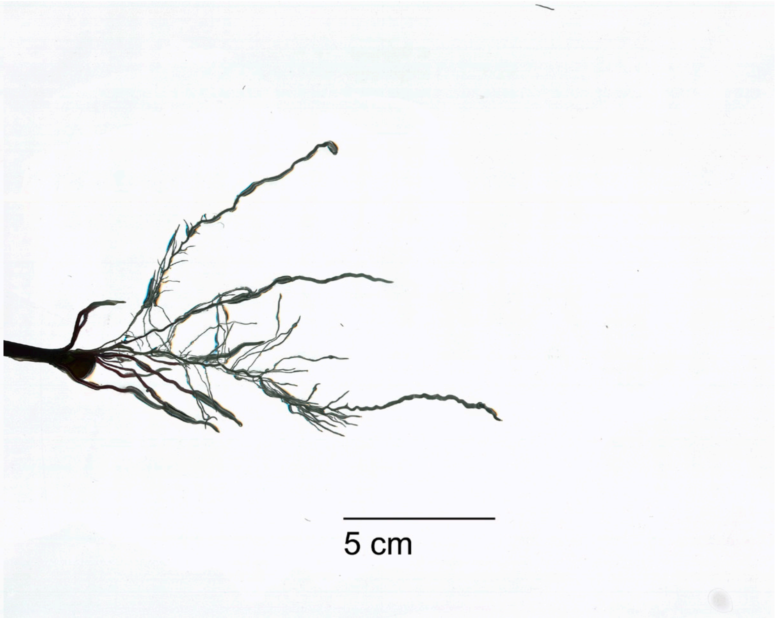

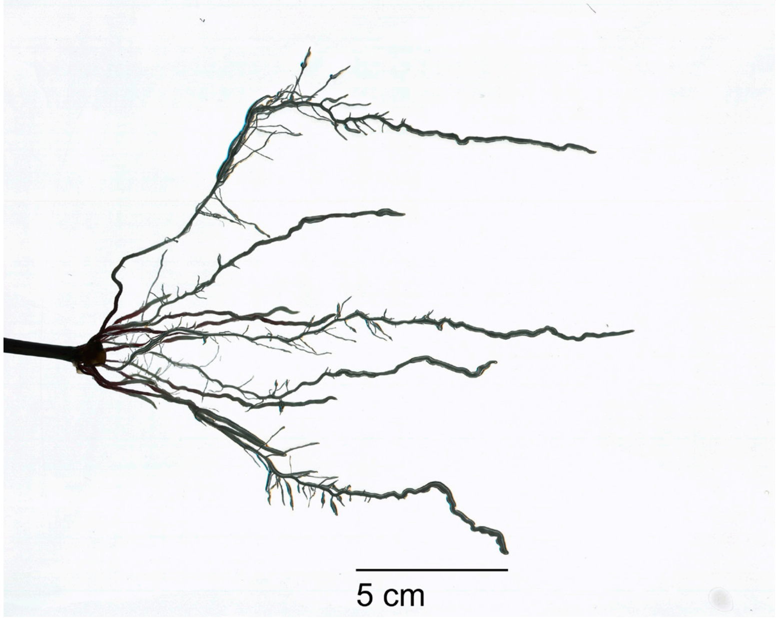

Supplement: Supplementary file 1 — Additional file 1: Figure S1. Scanned roots of seven maize genotypes grown in the glass bead-semi hydroponic system. Three images representative of each genotype are shown. The roots belong to plants grown 15 days after planting and after exudates were collected. [file 13007_2022_856_MOESM1_ESM.pdf]
