# Supplementary figures and images for: A glass bead semi-hydroponic system for intact maize root exudate analysis and phenotyping
Source: Plant Methods. 2022 Mar 5;18:25. doi: 10.1186/s13007-022-00856-4 (PMC8897885; doi:10.1186/s13007-022-00856-4)

**Ames 12734**

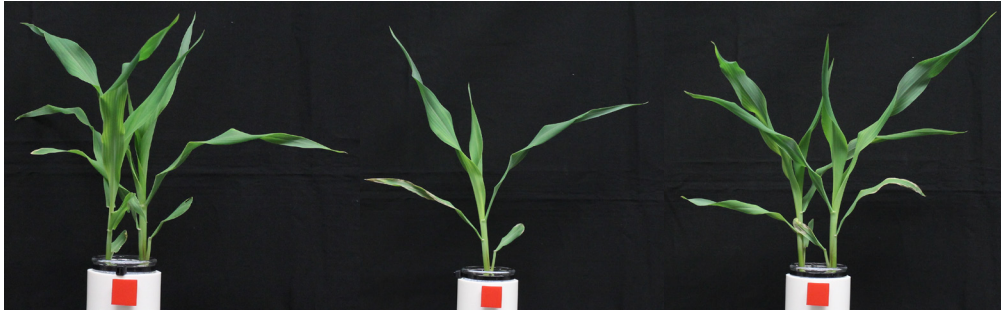

**Ames 20140**

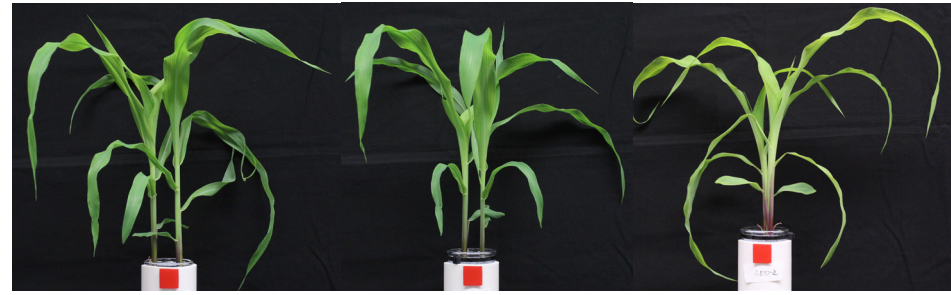

**Ames 20190**

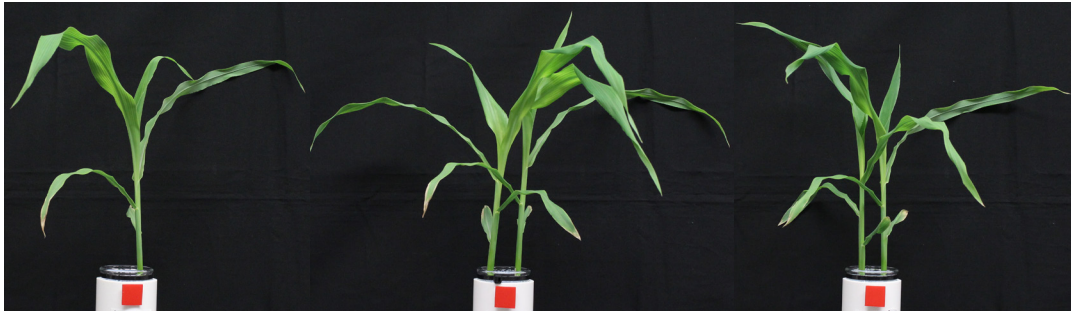

**Ames 27171**

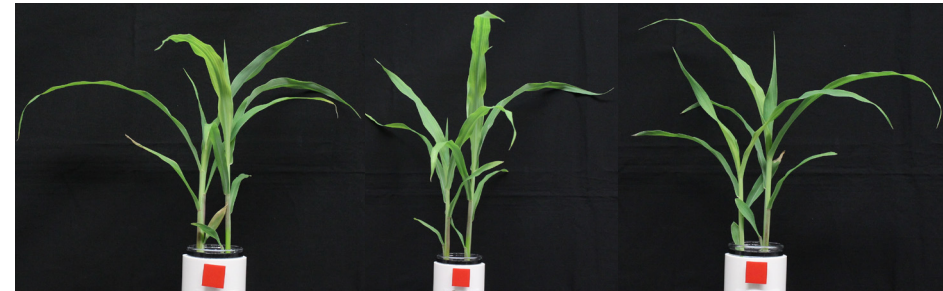

**Cize 7**

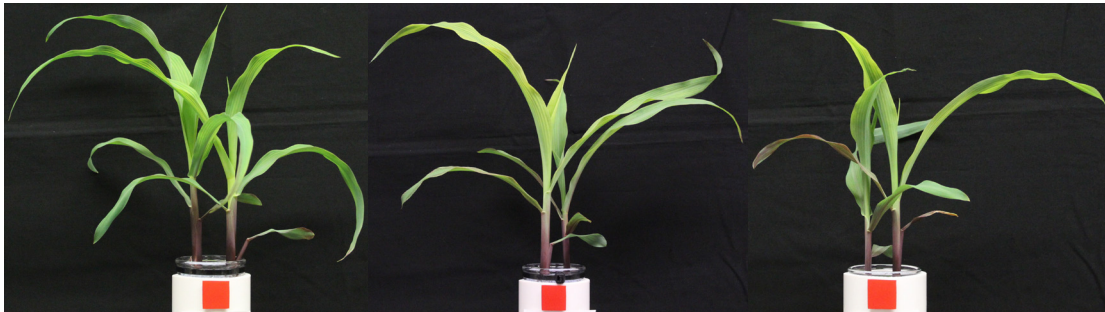

**NSL 22629**

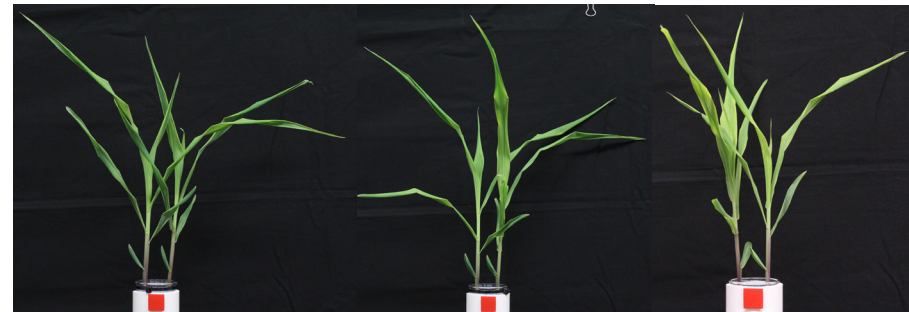

**PI 587154**

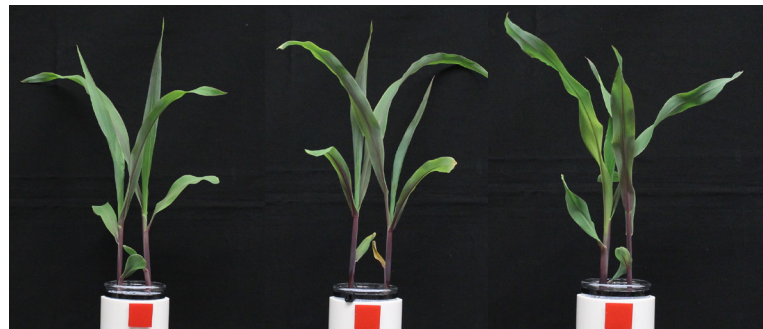

Supplement: Supplementary file 2 — Additional file 2: Figure S2. Images of shoots of seven genotypes grown in the glass bead-semi hydroponic system. Three representative pictures of each genotype are shown. The plants shown were grown for 15 days after planting. Red square = 1cm2. [file 13007_2022_856_MOESM2_ESM.pdf]

a

ng/g FRW

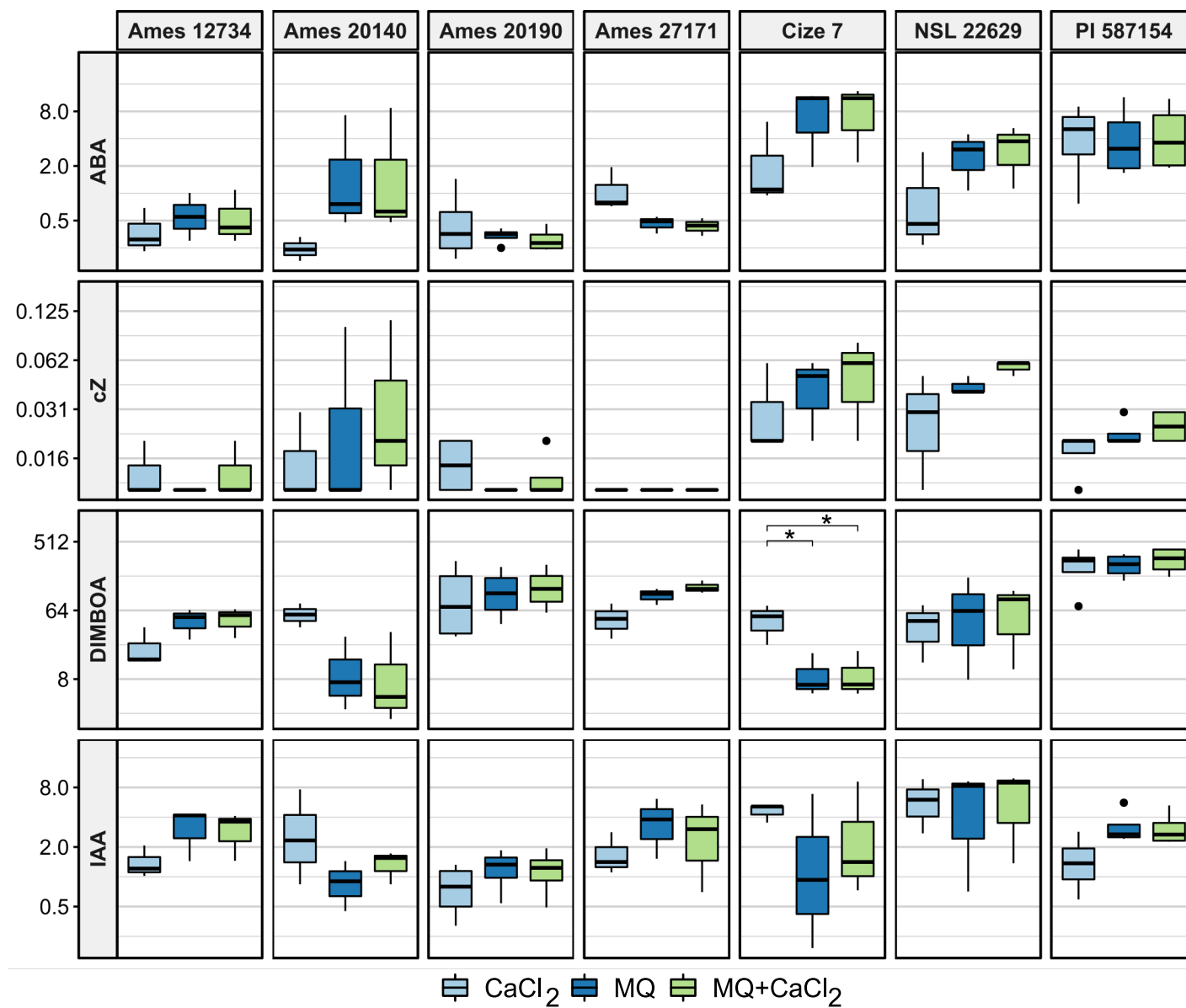

b

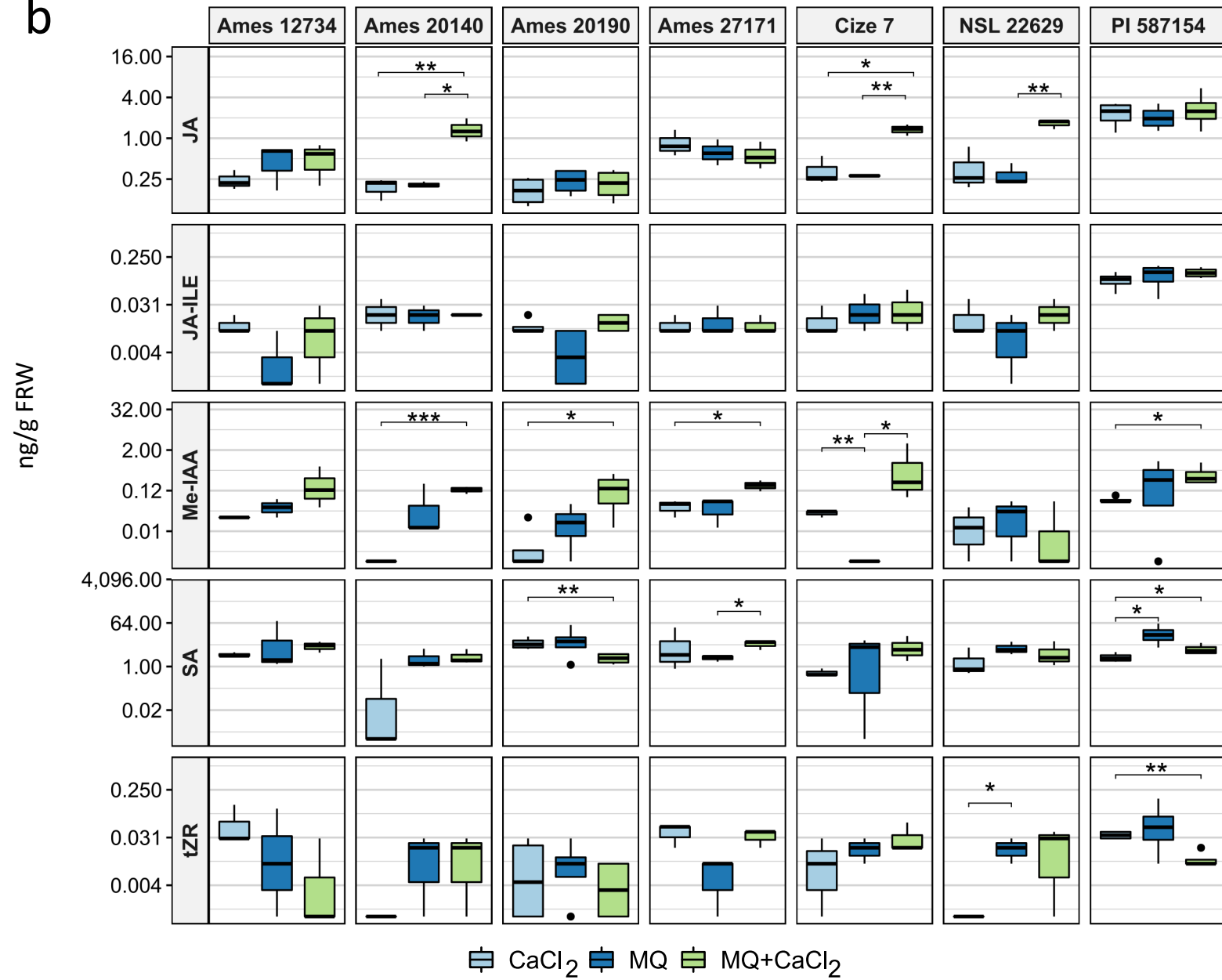

Supplement: Supplementary file 4 — Additional file 4: Figure S4. Differences in the concentration of phytohormones and DIMBOA detected in exudates collected with 1 mM CaCl2 and MQ in seven genotypes. Analysis of the effect of CaCl2 and MCX-SPE clean-up in the recovery of exudates. Different compounds are shown in two panels. a ABA (abscisic acid), cZ (cis-Zeatin), DIMBOA (2,4-dihydroxy-7-methoxy-1,4-benzoxazin-3-one), IAA (indole-3-acetic acid). b JA (jasmonic acid), JA-Ile (jasmonic acid-isoleucine, Me-lIAA (methyl- indole-3-acetic acid), SA (salicylic acid), tZR (trans-zeatin riboside). FRW (Fresh root weight). T test, each pair, only significant differences shown *p ≤ 0.05, **p ≤ 0.01. Cize 7, Ames 12734, Ames 20140, Ames 27171, NSL 22629 n = 3; Ames 20190, PI 57154 n = 4. Boxplot: Box, interquartile range (IQR); line inside the box, median; end of the box, upper (Q3) and lower (Q1) quartiles; dots beyond the extreme lines show potential outliers. [file 13007_2022_856_MOESM4_ESM.pdf]

a

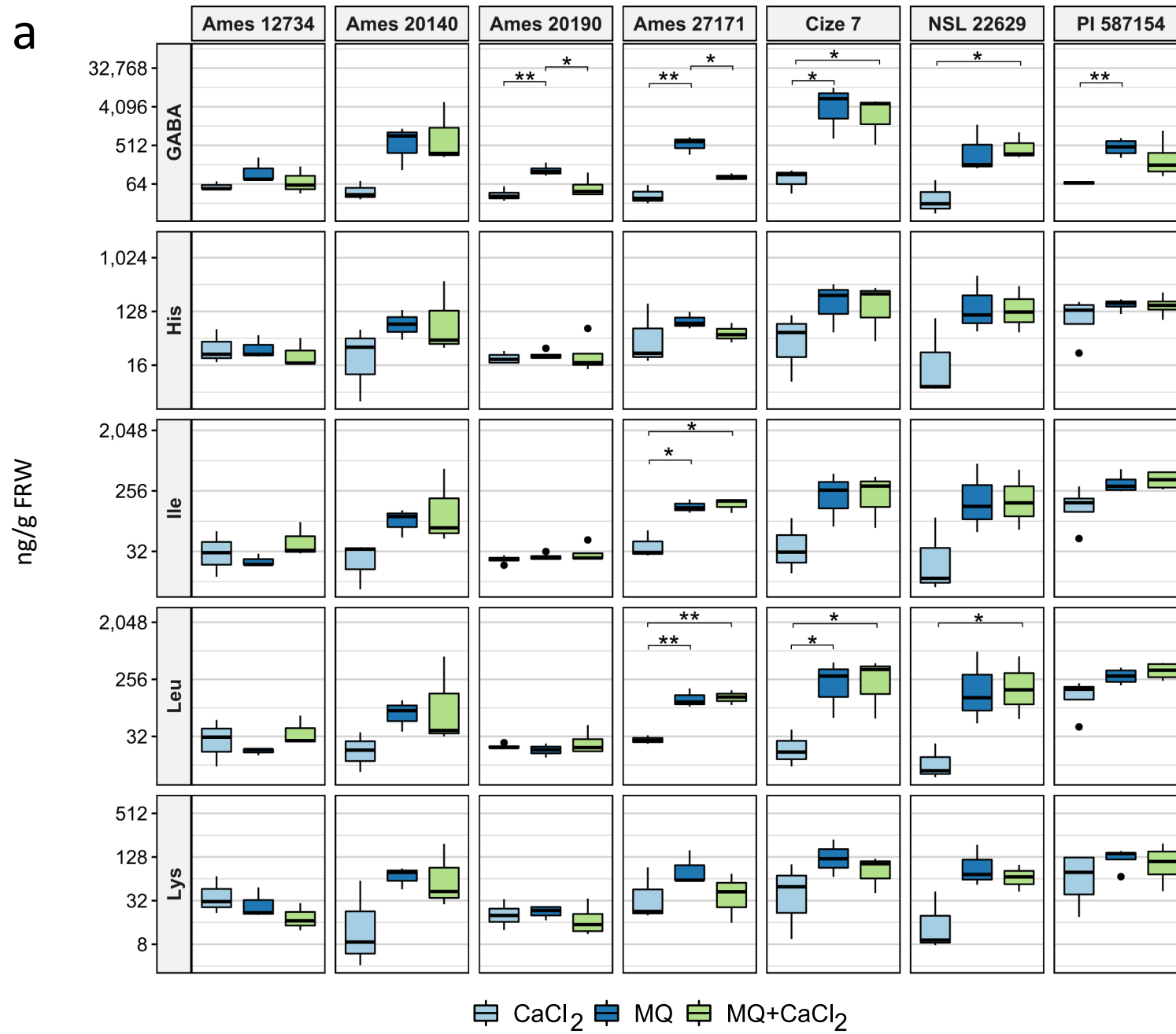

**b**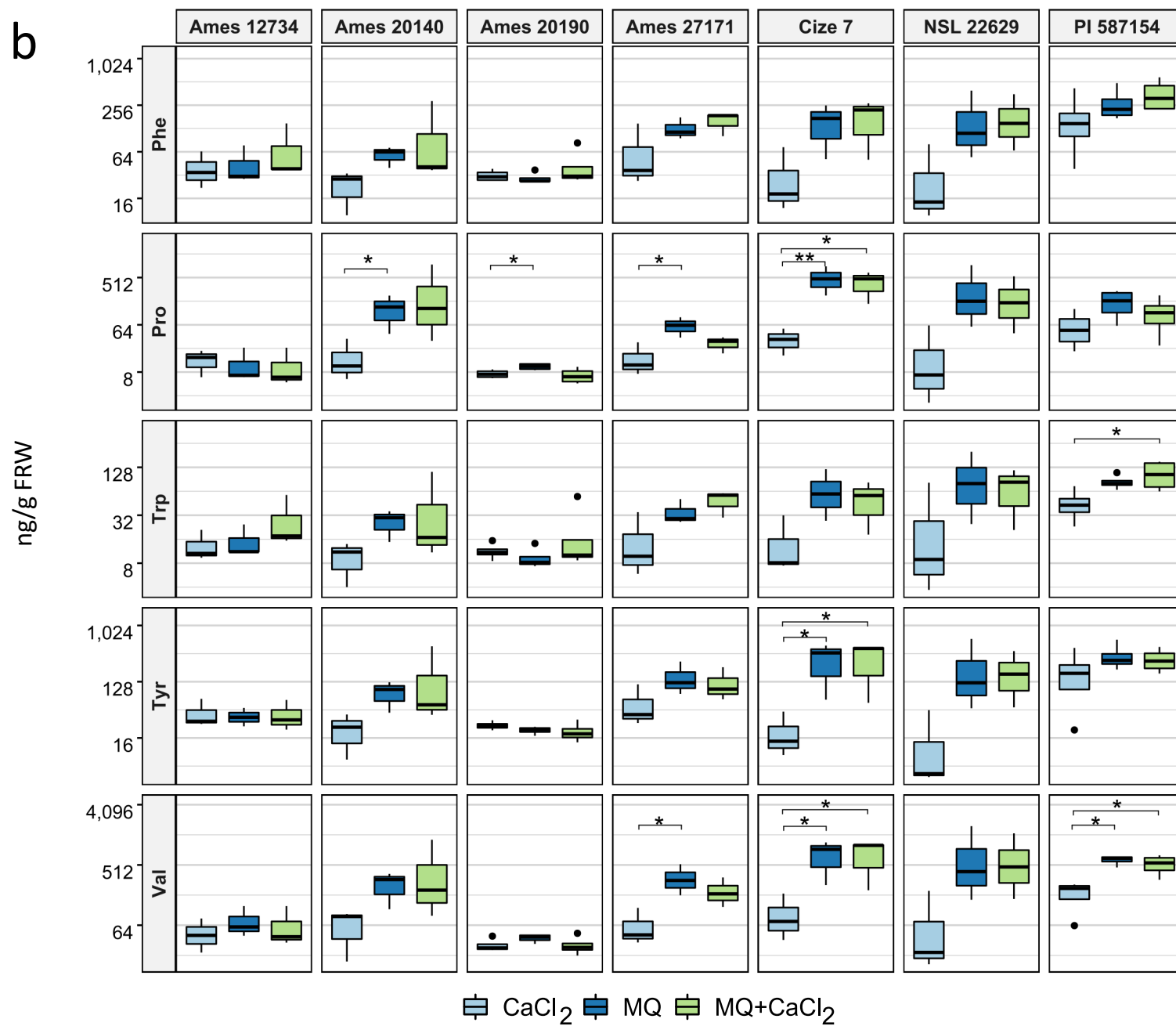

Supplement: Supplementary file 5 — Additional file 5: Figure S5. Differences in the concentration of the group 1 of amino acids detected in exudates collected with 1 mM CaCl2 and MQ in seven genotypes. Analysis of the effect of CaCl2 and MCX-SPE clean-up in the recovery of exudates. Different compounds are shown in two panels. a GABA (Gamma aminobutyric acid), His (histidine), Ile (isoleucine), Leu (leucine), Lys (lysine). b Phe (phenylalanine), Pro (proline), Trp (tryptophan), Tyr (tyrosine), Val (valine). FRW (Fresh root weight). T test, each pair, only significant differences shown *p ≤ 0.05, **p ≤ 0.01. Cize 7, Ames 12734, Ames 20140, Ames 27171, NSL 22629 n = 3; Ames 20190, PI 57154 n = 4. Boxplot: Box, interquartile range (IQR); line inside the box, median; end of the box, upper (Q3) and lower (Q1) quartiles; dots beyond the extreme lines show potential outliers. [file 13007_2022_856_MOESM5_ESM.pdf]

a

ng/g FRW

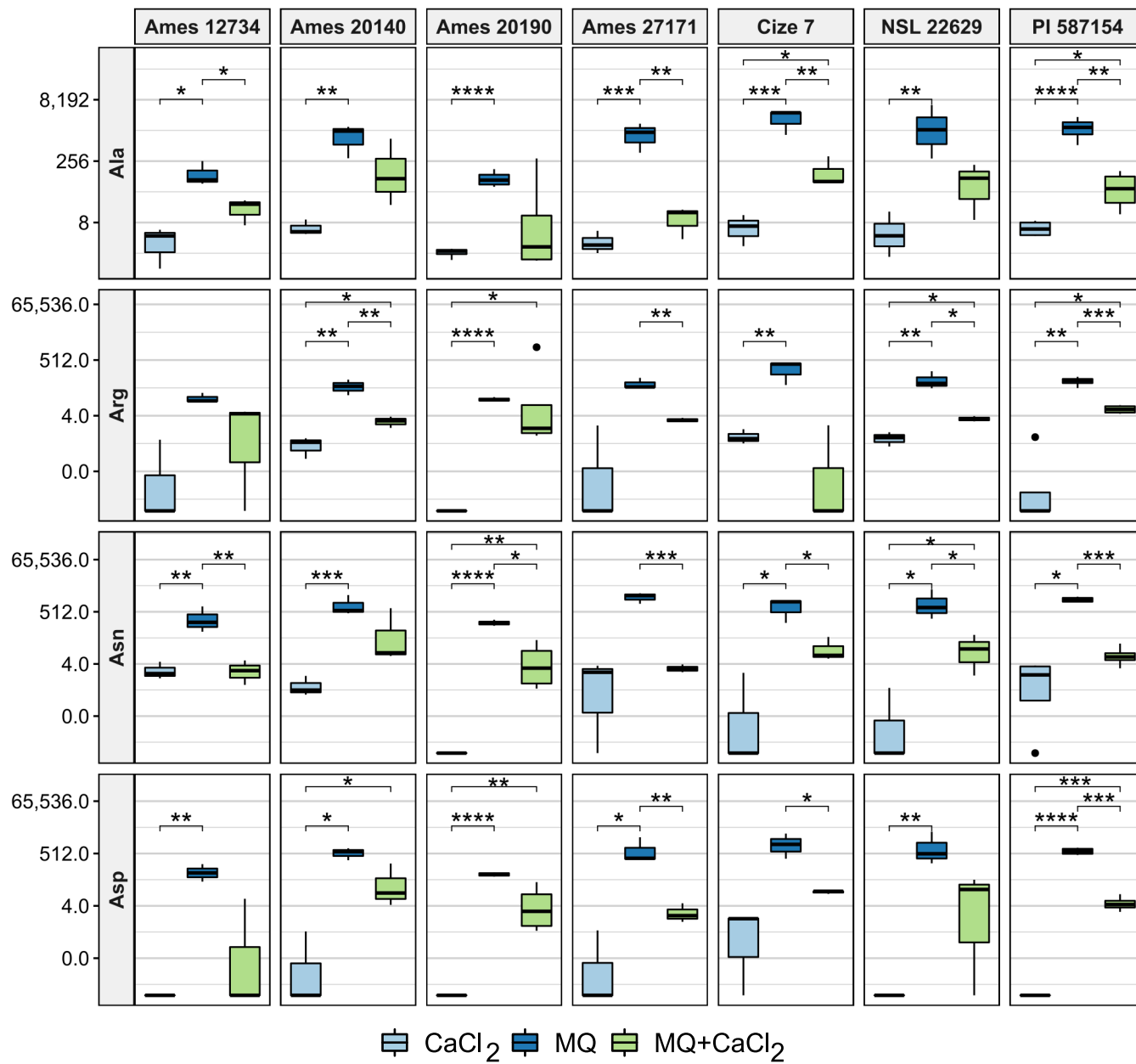

b

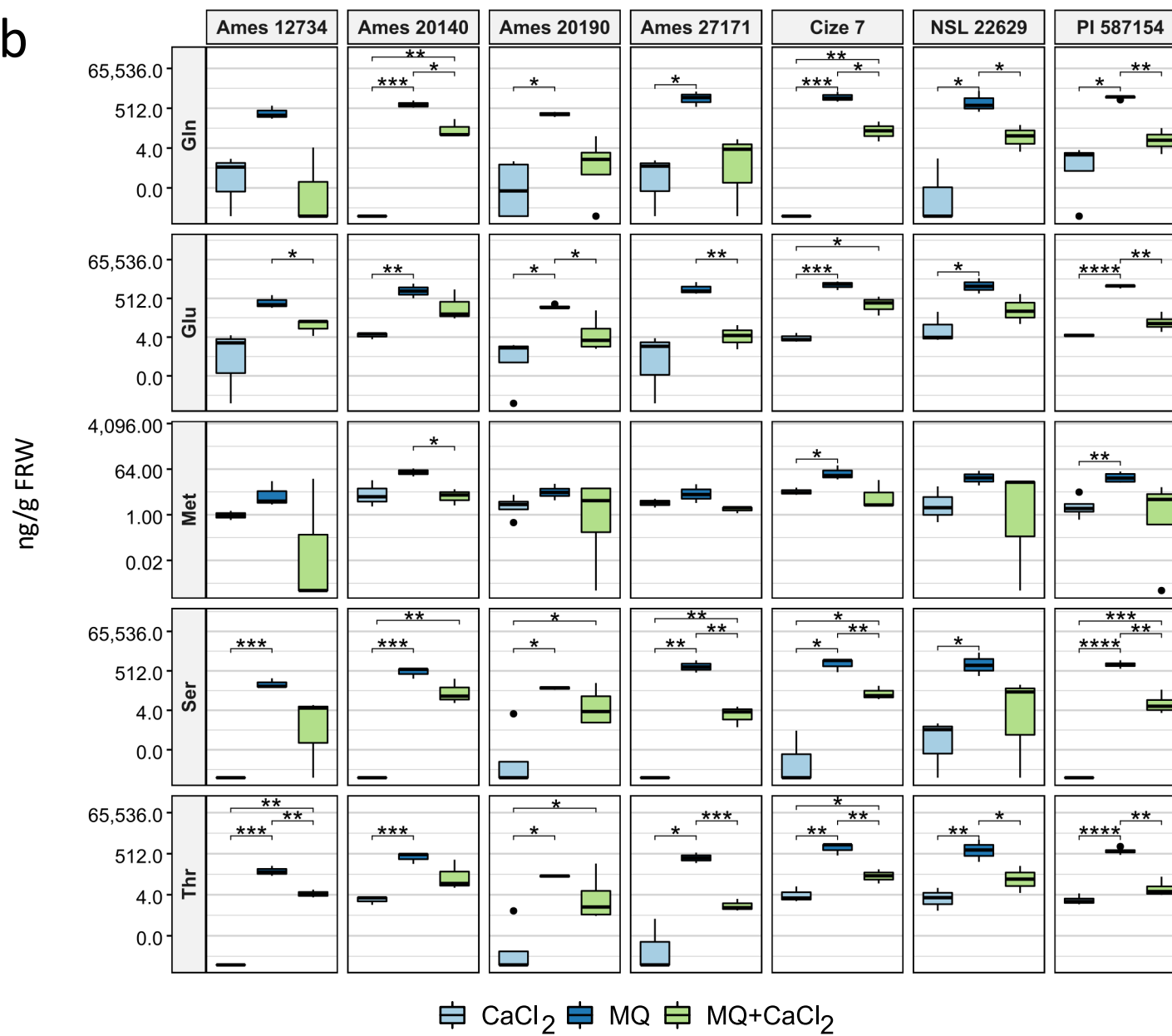

Supplement: Supplementary file 6 — Additional file 6: Figure S6. Differences in the concentration of the group 2 of amino acids detected in exudates collected with 1 mM CaCl2 and MQ in seven genotypes. Analysis of the effect of CaCl2 and MCX-SPE clean-up in the recovery of exudates. Different compounds are shown in two panels. a Ala (alanine), Arg (arginine), Asn (asparagine), Asp (aspartic acid). b Gln (glutamine), Glu (glutamic acid), Met (methionine), Ser (serine), Thr (threonine). FRW (Fresh root weight). T test, each pair, only significant differences shown *p ≤ 0.05, **p ≤ 0.01. Cize 7, Ames 12734, Ames 20140, Ames 27171, NSL 22629 n = 3; Ames 20190, PI 57154 n = 4. Boxplot: Box, interquartile range (IQR); line inside the box, median; end of the box, upper (Q3) and lower (Q1) quartiles; dots beyond the extreme lines show potential outliers. [file 13007_2022_856_MOESM6_ESM.pdf]

**a** $\mu\text{g/g FRW}$ 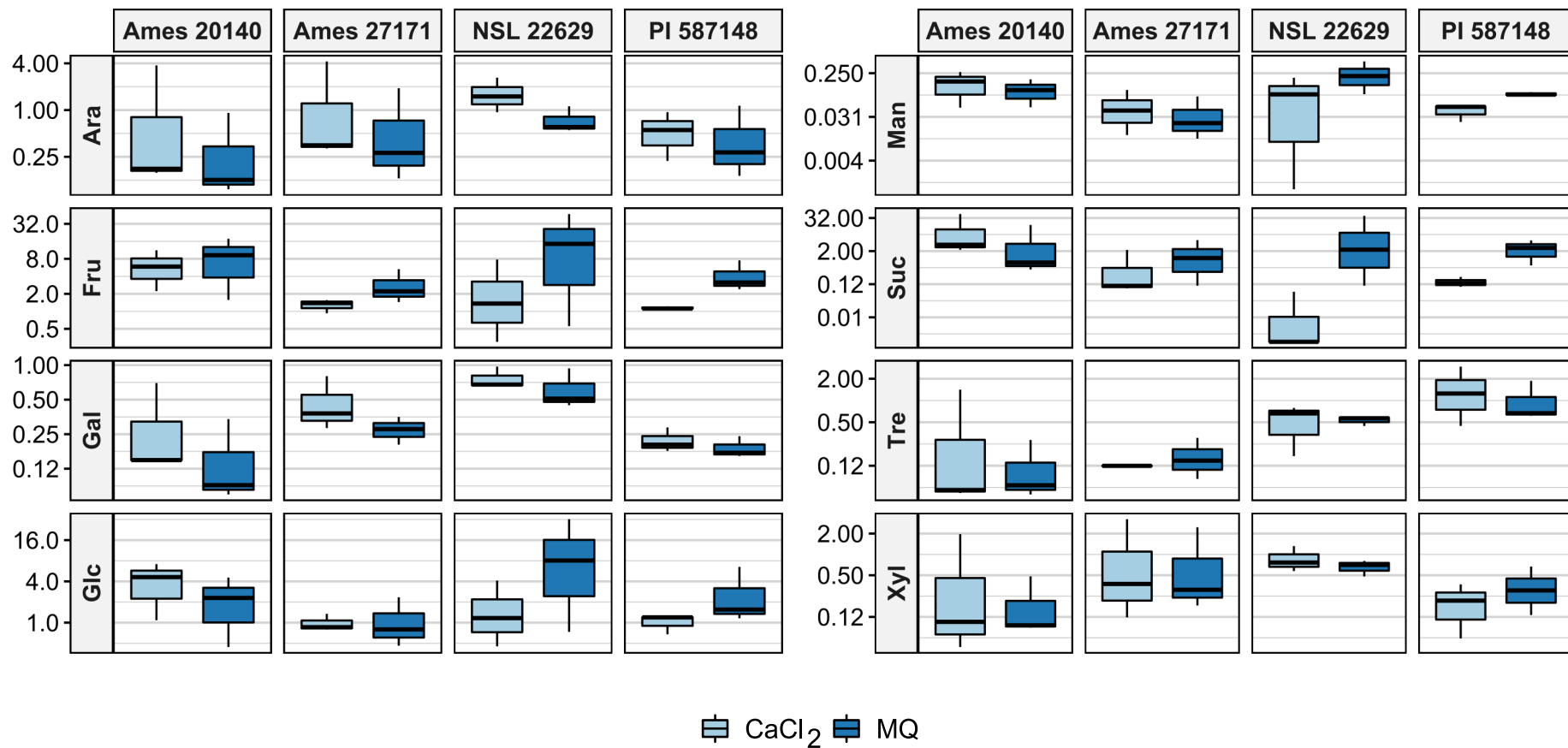

**b**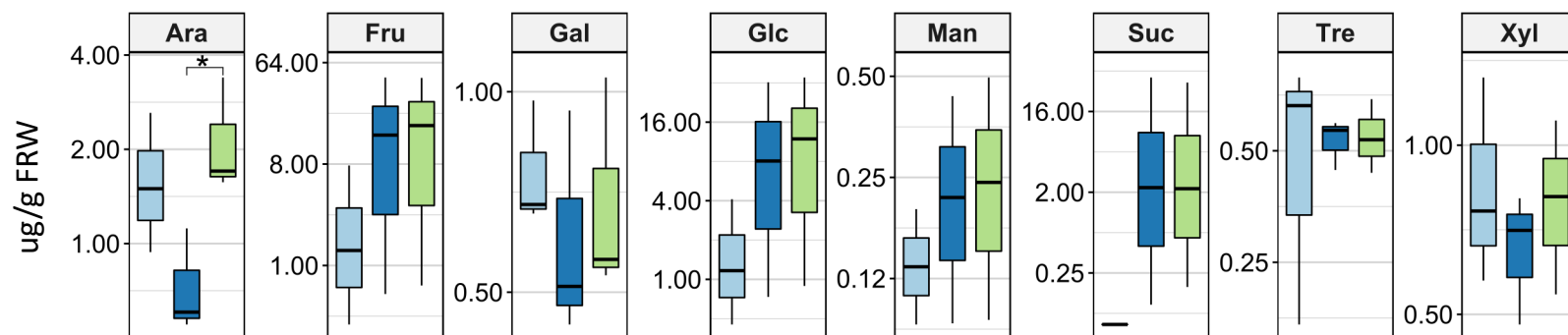**c**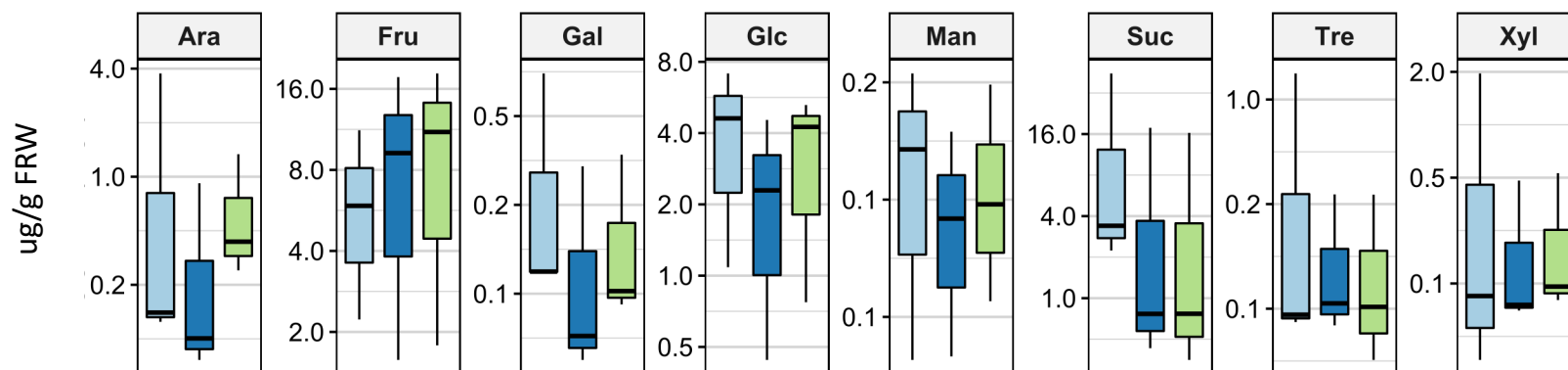

$\text{CaCl}_2$  MQ MQ+ $\text{CaCl}_2$

Supplement: Supplementary file 7 — Additional file 7: Figure S7. Differences in the concentration of sugars detected in exudates collected with 1 mM CaCl2 and MQ among genotypes. a Sugar content in four genotypes. b Sugar content in the root exudates of genotype NSL 22629 (n = 3), effect of CaCl2 on the recovery of sugars. c Sugar content in the root exudates of genotype Ames 20140 (n = 3), effect of CaCl2 on the recovery of sugars. Ara (arabinose), Fru (fructose), Gal (galactose), Glc (glucose), Man (mannose), Suc (sucrose), Tre (trehalose), Xyl (xylose). FRW (Fresh root weight). T test, each pair, *p ≤ 0.05, only significant differences shown. Boxplot: Box, interquartile range (IQR); line inside the box, median; end of the box, upper (Q3) and lower (Q1) quartiles; dots beyond the extreme lines show potential outliers. [file 13007_2022_856_MOESM7_ESM.pdf]

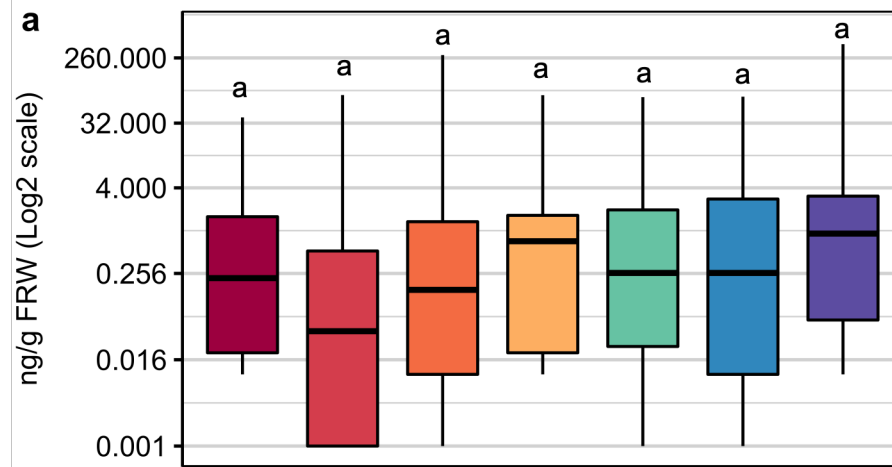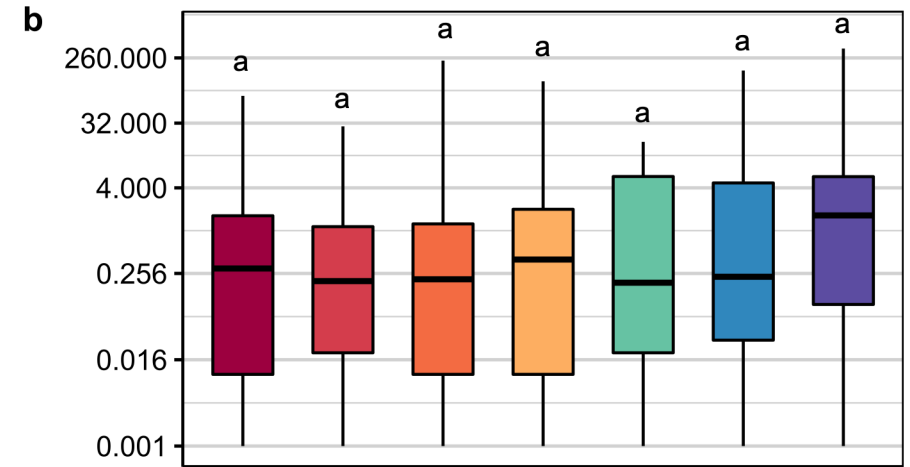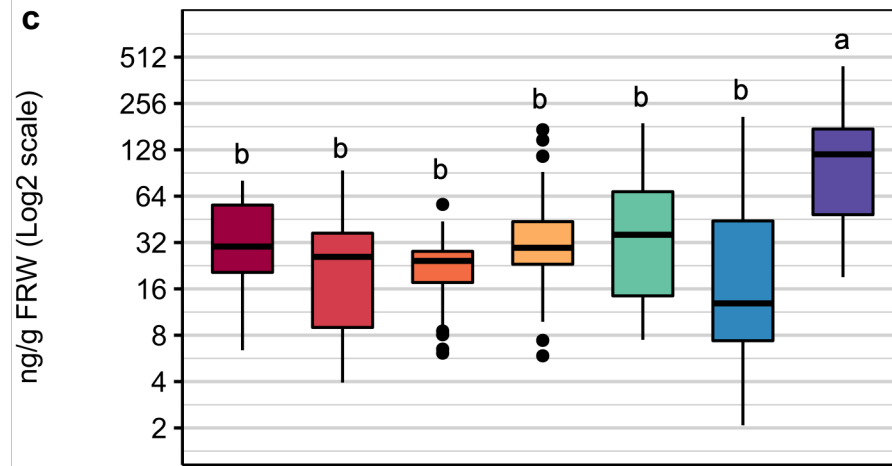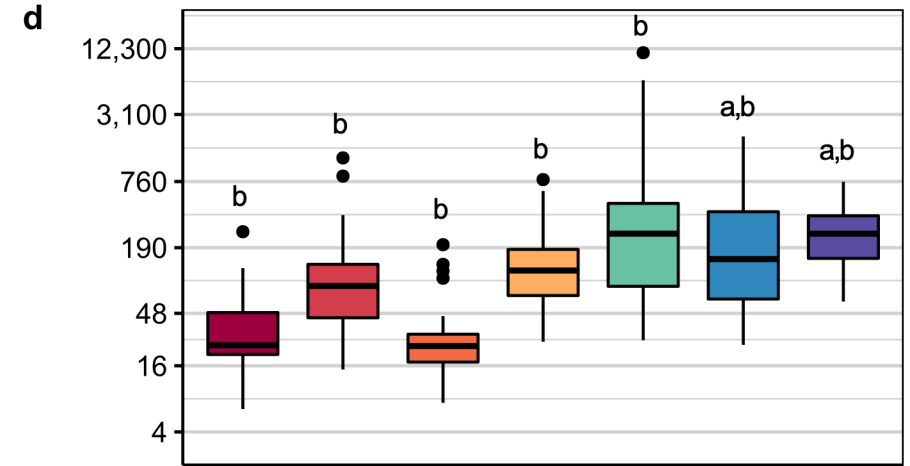

■ Ames 12734 
 ■ Ames 20140 
 ■ Ames 20190 
 ■ Ames 27171 
 ■ Cize 7 
 ■ NSL 22629 
 ■ PI 587154

Supplement: Supplementary file 8 — Additional file 8: Figure S8. Genotypic differences in the concentration of phytohormones and amino acids detected in exudates collected with MQ and 1 mM CaCl2. a Phytohormones detected in calcium chloride. b Phytohormones detected in MQ. c Amino acids detected in calcium chloride. d Amino acids detected in MQ. Statistical differences detected by All pairs Tukey–Kramer HSD, α = 0.05. Measurements with different letters within each graph are significantly different. Boxplot: Box, interquartile range (IQR); line inside the box, median; end of the box, upper (Q3) and lower (Q1) quartiles; dots beyond the extreme lines show potential outliers. [file 13007_2022_856_MOESM8_ESM.pdf]
