# Supplementary material for: A glass bead semi-hydroponic system for intact maize root exudate analysis and phenotyping
Source: Plant Methods. 2022 Mar 5;18:25. doi: 10.1186/s13007-022-00856-4 (PMC8897885; doi:10.1186/s13007-022-00856-4)
Supplement: Supplementary file 3 — Additional file 3: Figure S3. Comparison of root morphology between maize plants growing in the glass bead semi-hydroponic system and hydroponics. a Root morphology of four genotypes of corn grown in glass bead semi-hydroponic system and hydroponics. Different types of roots are explained in the images of the genotype PI 587154 as an example. b Two corn genotypes grown in different substrates: glass bead semi-hydroponic, hydroponics, sand, and soil. c Close-up to selected images to illustrate the presence of root hairs in the plants growing in the glass bead semi-hydroponic system but not when the plants are grown using hydroponics. [file 13007_2022_856_MOESM3_ESM.pdf]

a

PI 587154 glass bead semi-hydroponic **PI 587154 hydroponics**

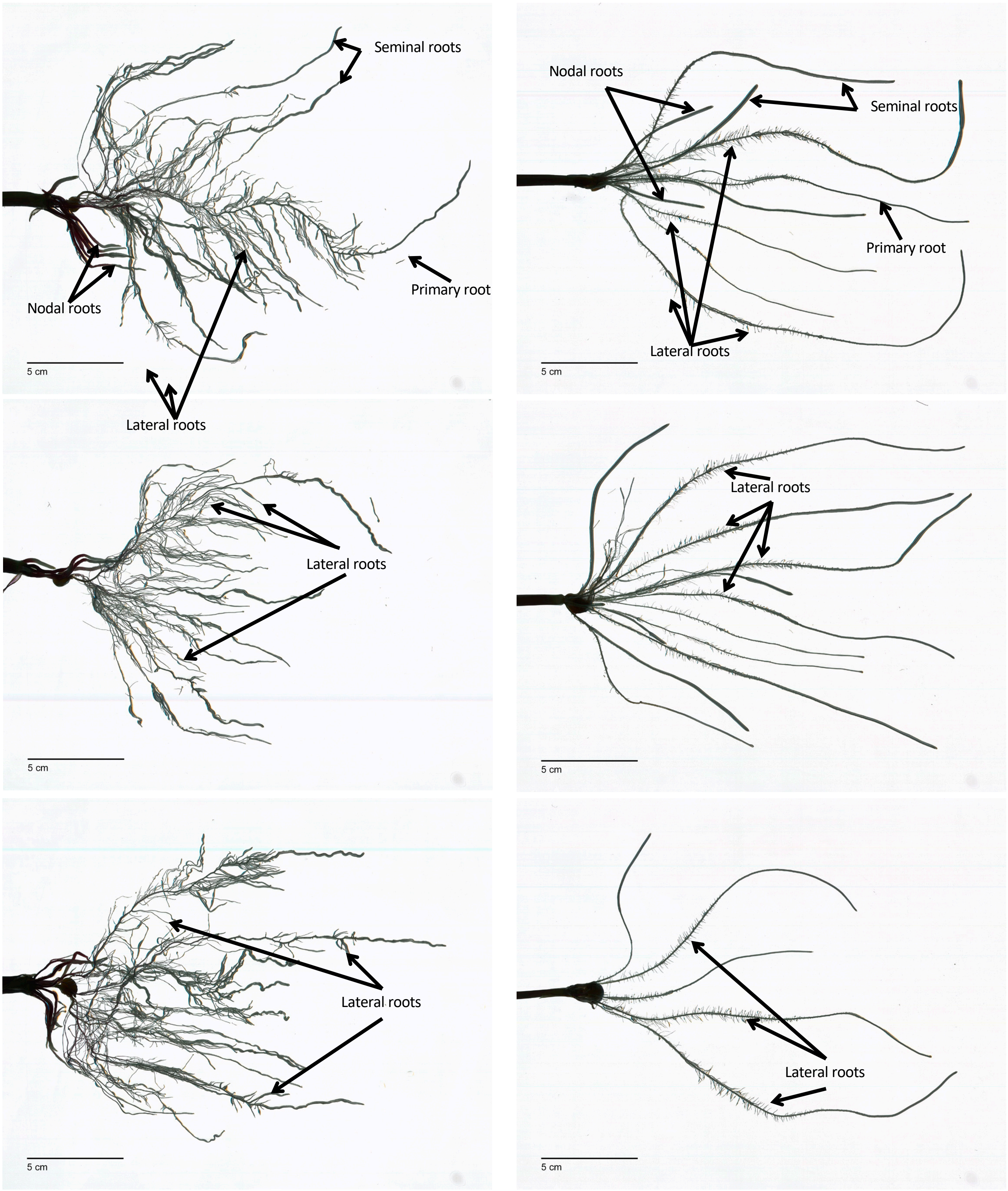

a

B73 glass bead semi-hydroponic | **B73 hydroponics**

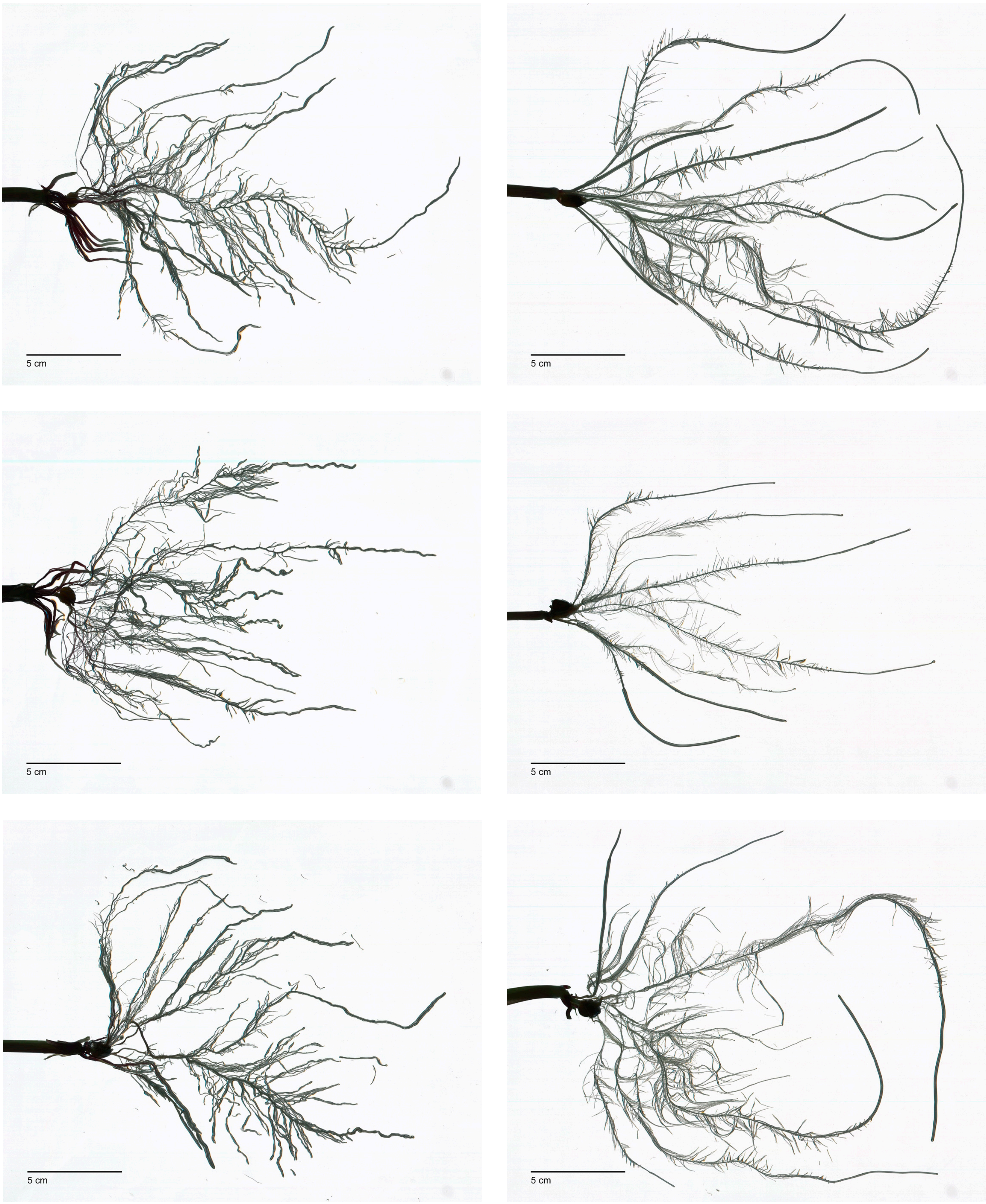

a

Ames 27136 glass bead semi-hydroponic | **Ames 27136 hydroponics**

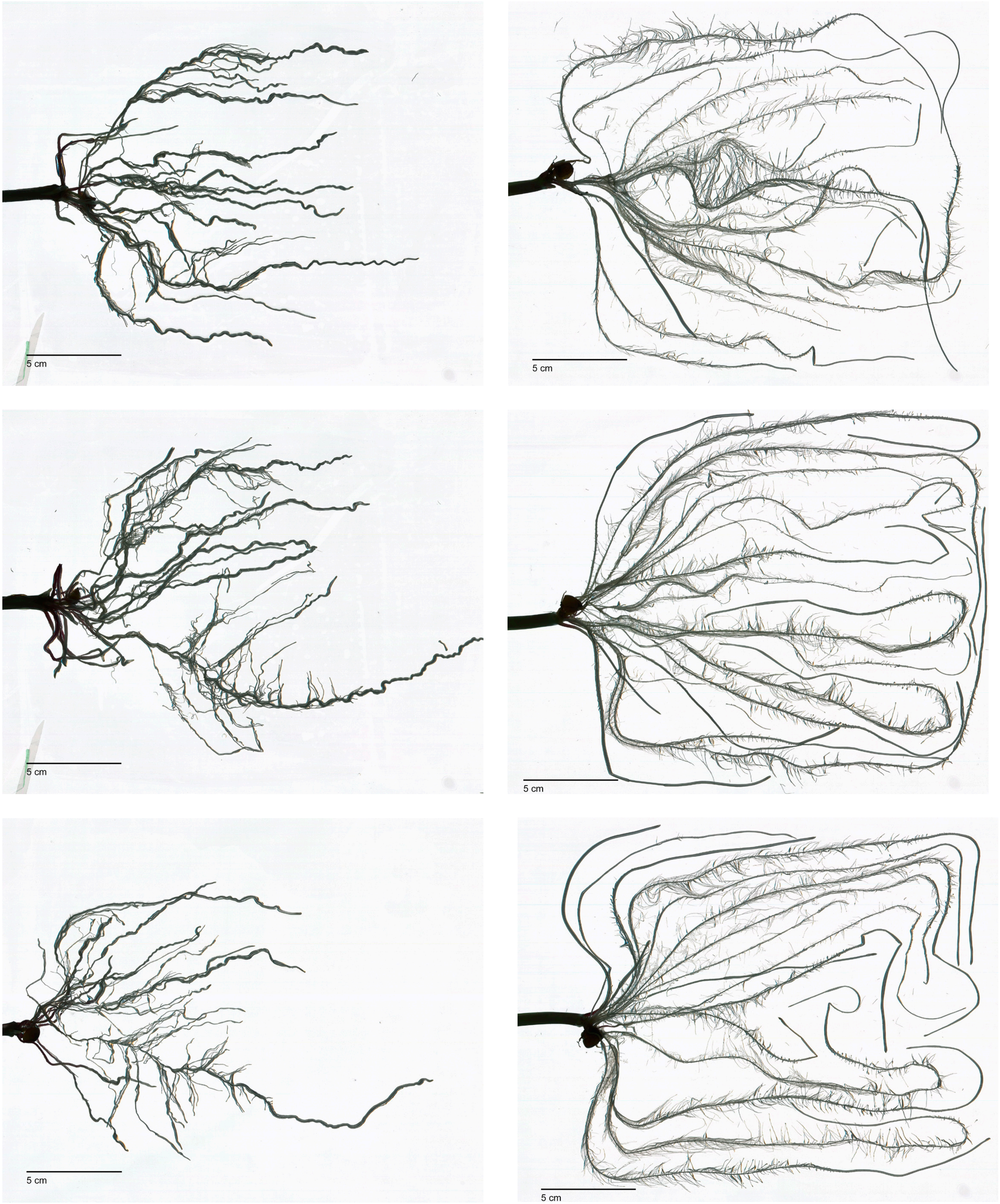

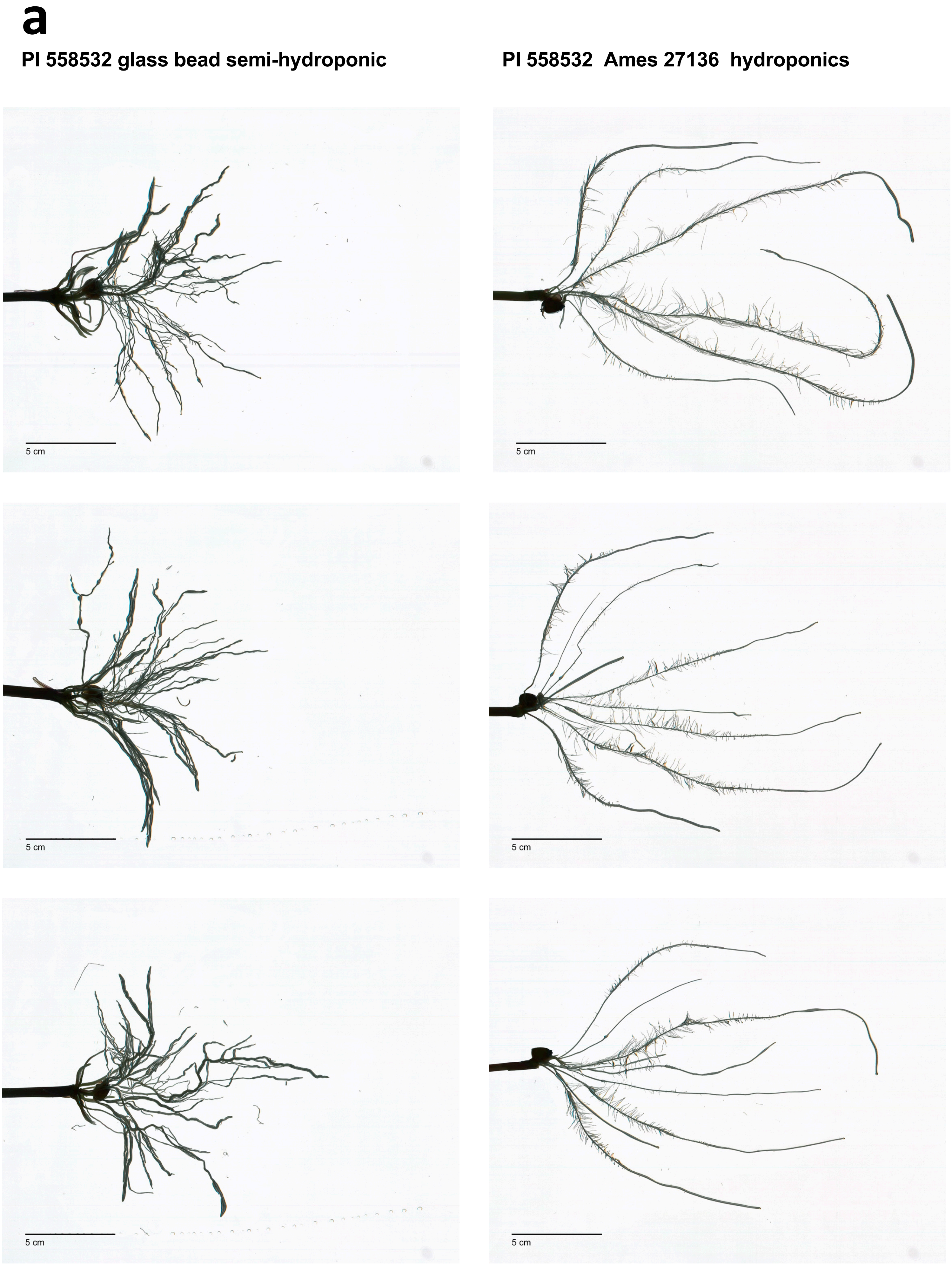
a
PI 558532 glass bead semi-hydroponic
PI 558532 Ames 27136 hydroponics
5 cm
5 cm
5 cm
5 cm
5 cm
5 cm

b

Additional file 3. Comparison of root morphology between maize plants growing in the glass bead-semi hydroponic system and hydroponics. b. Two corn genotypes grown in different substratum, glass bead-semi hydroponic, hydroponics, sand, and soil.

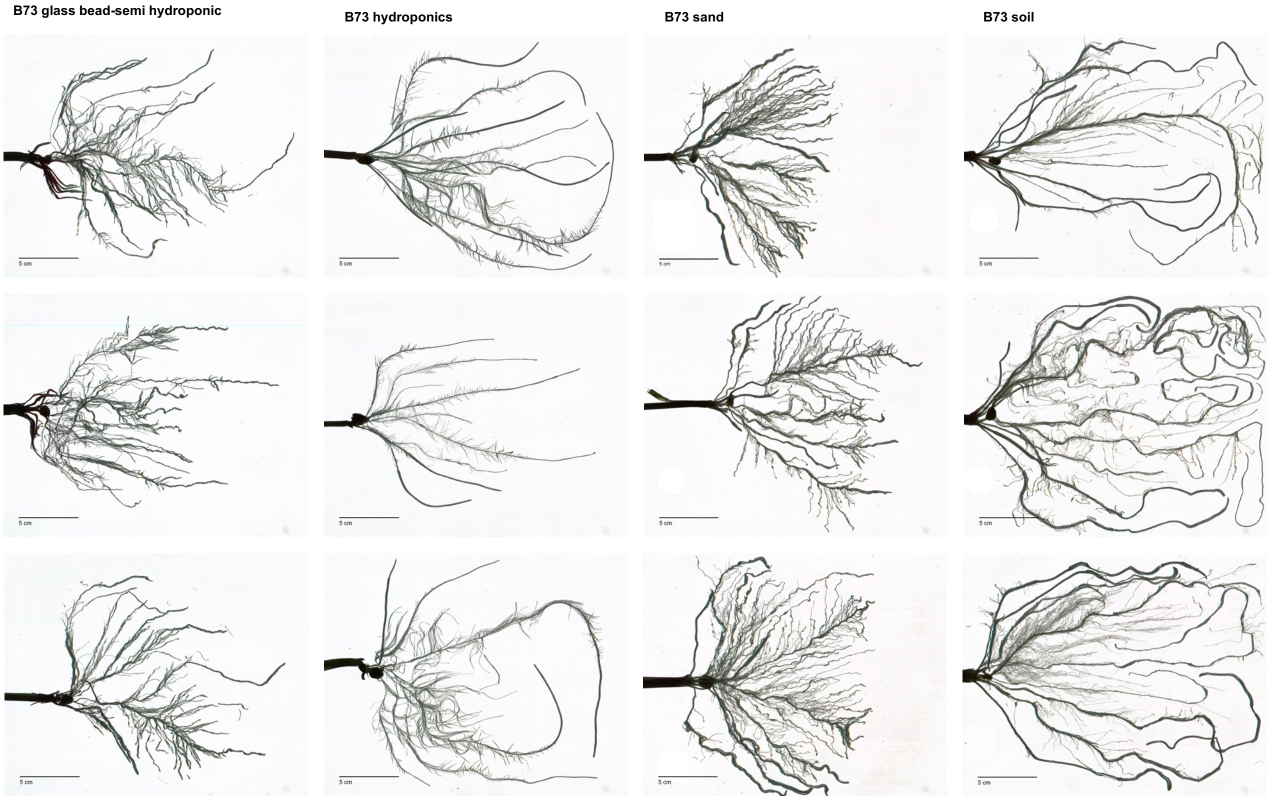

b

Additional file 3. Comparison of root morphology between maize plants growing in the glass bead-semi hydroponic system and hydroponics.
b. Two corn genotypes grown in different substratum, glass bead-semi hydroponic, hydroponics, sand, and soil.

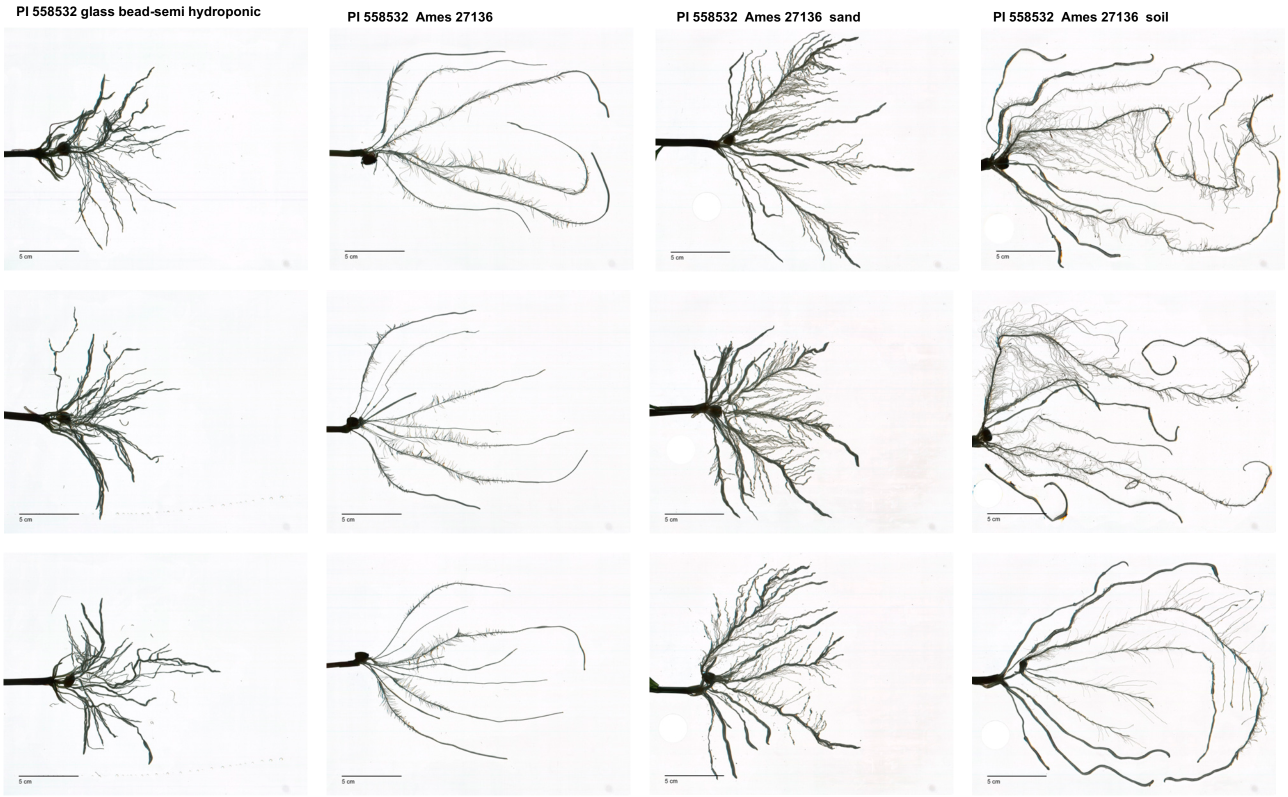

c

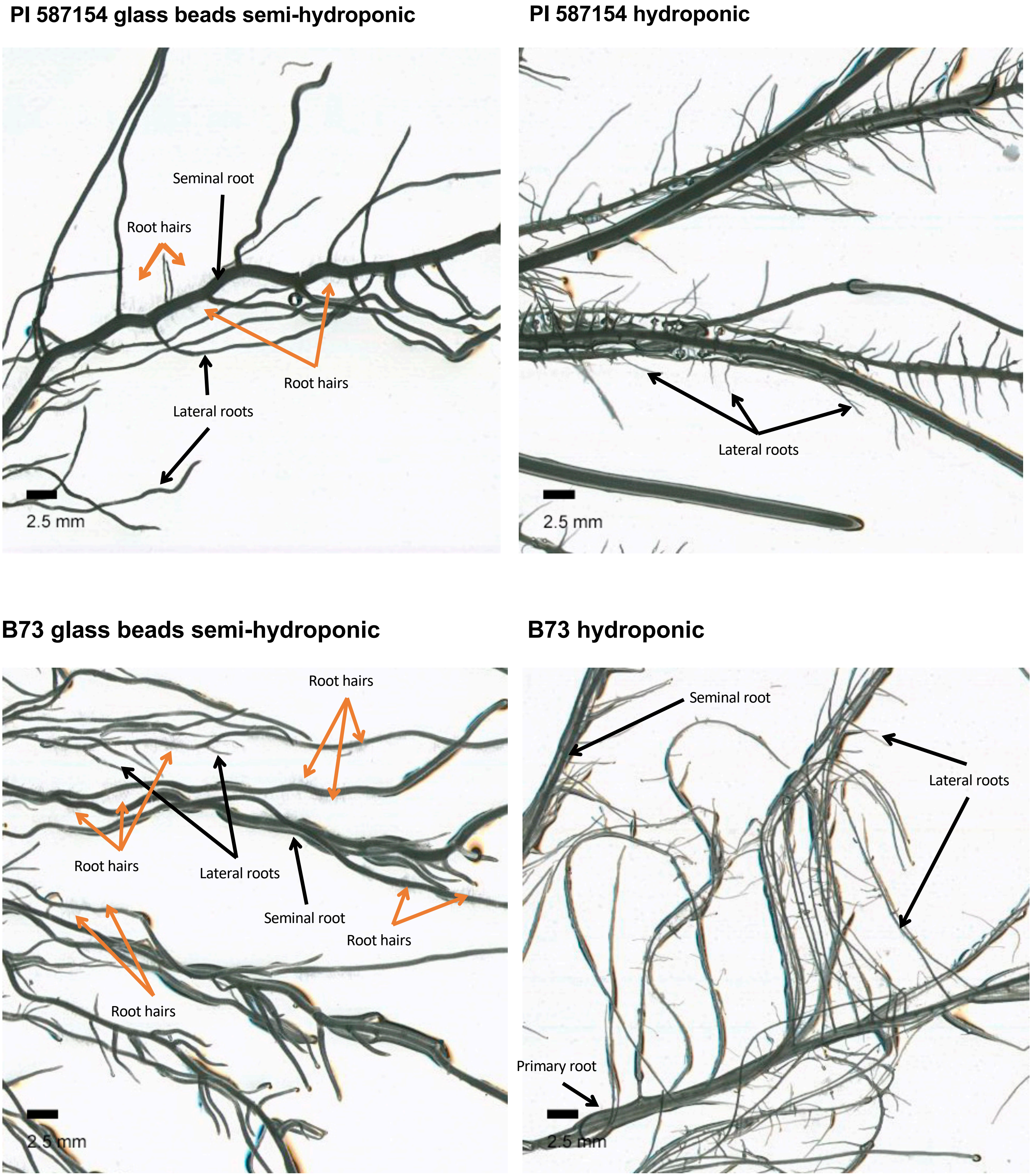
PI 587154 glass beads semi-hydroponic
Seminal root
Root hairs
Root hairs
Lateral roots
2.5 mm
PI 587154 hydroponic
Lateral roots
2.5 mm
B73 glass beads semi-hydroponic
Root hairs
Root hairs
Lateral roots
Seminal root
Root hairs
Root hairs
2.5 mm
B73 hydroponic
Seminal root
Lateral roots
Primary root
2.5 mm

c

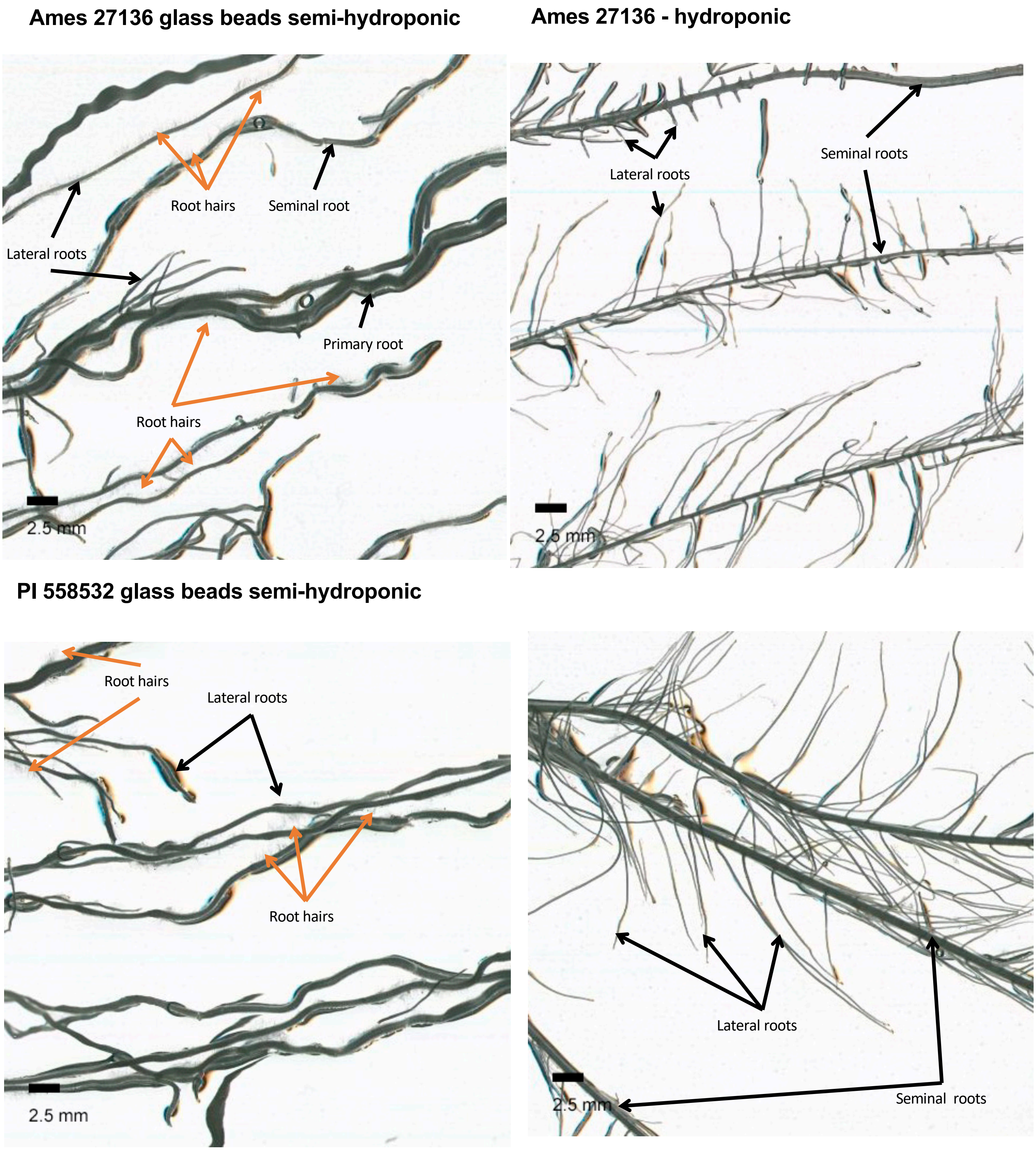
Ames 27136 glass beads semi-hydroponic
Root hairs
Seminal root
Lateral roots
Primary root
Root hairs
2.5 mm
Ames 27136 - hydroponic
Lateral roots
Seminal roots
2.5 mm
PI 558532 glass beads semi-hydroponic
Root hairs
Lateral roots
Root hairs
2.5 mm
Lateral roots
Seminal roots
2.5 mm
