# Supplementary material for: A glass bead semi-hydroponic system for intact maize root exudate analysis and phenotyping
Source: Plant Methods. 2022 Mar 5;18:25. doi: 10.1186/s13007-022-00856-4 (PMC8897885; doi:10.1186/s13007-022-00856-4)
Supplement: Supplementary file 9 — Additional file 9: Table S1. Comparison of the levels of amino acids, sugars and DIMBOA detected in this paper with those detected in previous studies. [file 13007_2022_856_MOESM9_ESM.pdf]

Additional file 9. Comparison of the levels of amino acids, sugars and DIMBOA detected in this paper with previous publications.

Reference	Growing method	Plant age	Collection time	Collection solution	Compounds quantified	Concentration range*	Unit**
This paper	Glas bead-semi-hydroponic	2 weeks old	2 hours	water	Amino Acids	6-11,000	ng/g FRW
					Sugars	0.01-40	µg/g FRW
					DIMBOA	0.003-0.35	µg/g FRW
				1 mM $CaCl_2$	Amino Acids	2-450	ng/g FRW
					Sugars	0.001-45	µg/g FRW
					DIMBOA	0.015-0.4	µg/g FRW
Fan et al, 2012 [50]	Axenic hydroponic	3-7 days old	Not indicated	water	Amino Acids	0.1-4	mg/g DW
					Sugars	1-12	mg/g DW
Lapie et al, 2019 [51]	Sand	6 weeks old	6 hours	water	Amino Acids	100-3,000	ng/g DRW
					Sugars	1-1,000	µg/g DRW
Carvalhais et al, 2011 [30]	Axenic hydroponic	Fourth-leaf stage – 2 weeks old	6 hours	water	Amino Acids	0.5-10	ng/g FW
Oburger et al, 2013 [32]	Hydroponic	8-9 weeks old	24 hours	water	Amino Acids	0.01-0.025	ng/g RDW
				0.5 mM $CaCl_2$	Amino Acids	0-0.08	ng/g RDW
	Rhizotron	8-9 weeks old	24 hours	water	Amino Acids	0.02-0.2	ng/g RDW
				0.5 mM $CaCl_2$	Amino Acids	0.05-0.4	ng/g RDW
	Soil	8-9 weeks old	24 hours	water	Amino Acids	0.02-0.04	ng/g RDW
Neal et al, 2012 [52]	Soil	7, 14 and 21 days old	7 hours	water	DIMBOA	0.005-0.02	mg/g FRW
Marti et al, 2013 [53]	Sand	10-12 days old	48 hours	Methanol/ water (1:1)	DIMBOA derivatives	1-4	mg/g dried root exudate

Notes:

* When values were not reported in tables, the range was estimated visually from the graphs. The range reported uses the lowest value and the highest value reported for any given amino acid or sugar.

FRW (fresh root weight), DRW (root dry weight), FW (fresh weight), DW, dry weight
