# Supplementary material for: A glass bead semi-hydroponic system for intact maize root exudate analysis and phenotyping
Source: Plant Methods. 2022 Mar 5;18:25. doi: 10.1186/s13007-022-00856-4 (PMC8897885; doi:10.1186/s13007-022-00856-4)
Supplement: Supplementary file 10 — Additional file 10: Table S2. Phytohormones not detected in root exudates. Percentage recovery before and after normalization. Percentages of the phytohormones after SPE. Recoveries were calculated before and after normalization using the internal standards D2GA1 for GAs 1, 3, 4, 8, 9, 12, 19, 20 and 53, D5IAA for IAA-Ala, IAA-Asp and IAA-Trp, D2JA for OPDA, D5tZ for tZ. Coefficient of variation (CV) are also included. [file 13007_2022_856_MOESM10_ESM.pdf]

Additional file 10. Phytohormones not detected in root exudates. Percentage recovery before and after normalization. Percentages of the phytohormones after SPE. Recoveries were calculated before and after normalization using the internal standards D2GA1 for GAs 1, 3, 4, 8, 9, 12, 19, 20 and 53, D5IAA for IAA-Ala, IAA-Asp and IAA-Trp, D2JA for OPDA, D5tZ for tZ. Coefficient of variation (CV) are also included.

	% recovery before normalization		% recovery after normalization		CV in %	
	SPE-$CaCl_2$	SPE-MQ	SPE-CaCl2	SPE-MQ	SPE-CaCl2	SPE-MQ
**GA1**	86.2	87.1	102.7	101.4	2.6	4.4
**GA3**	81.3	83	96.7	96.5	6.8	2.4
**GA4**	92	91.3	109.4	106.2	3.9	0.6
**GA8**	96.7	95.7	115	111.3	3.4	5.1
**GA9**	83.8	81	99.8	94.2	2.8	3.9
**GA12**	60.6	51.4	72	59.9	4.6	12.7
**GA19**	91.5	92	108.7	107.1	4	4.7
**GA20**	89.9	90.4	106.9	105.1	4.1	3.2
**GA53**	91.4	88.5	108.7	102.9	3.6	1.9
**D2GA1**	83.9	85.8	100	100	4.3	3.5
**IAA-Ala**	73.1	77.4	157.8	141.2	4.1	5.8
**IAA-Asp**	74.8	76.1	161.7	138.8	3	1
**IAA-Trp**	73.6	76.6	159.1	140	3	2.2
**D5IAA**	46.7	54.9	100	100	12.1	5
**OPDA**	28.4	21	54.2	51.5	9	15.8
**D2JA**	53.1	41.3	100	100	12.6	12.2
**tZ**	70.6	71.5	95.7	88.2	3.3	3.6
**D5tZ**	73.8	81.4	100	100	4.4	7.4
